# Polymerization and Applications of Poly(methyl methacrylate)–Graphene Oxide Nanocomposites: A Review

**DOI:** 10.1021/acsomega.2c04483

**Published:** 2022-12-15

**Authors:** Muhammad Naziff Ahamad Said, Nurul Anis Hasbullah, Muhammad Ridhwan Hafiz Rosdi, Muhamad Sharan Musa, Arjulizan Rusli, Azlan Ariffin, Mohamad Danial Shafiq

**Affiliations:** School of Materials and Mineral Resources Engineering, Universiti Sains Malaysia, Engineering Campus, 14300Nibong Tebal, Penang, Malaysia

## Abstract

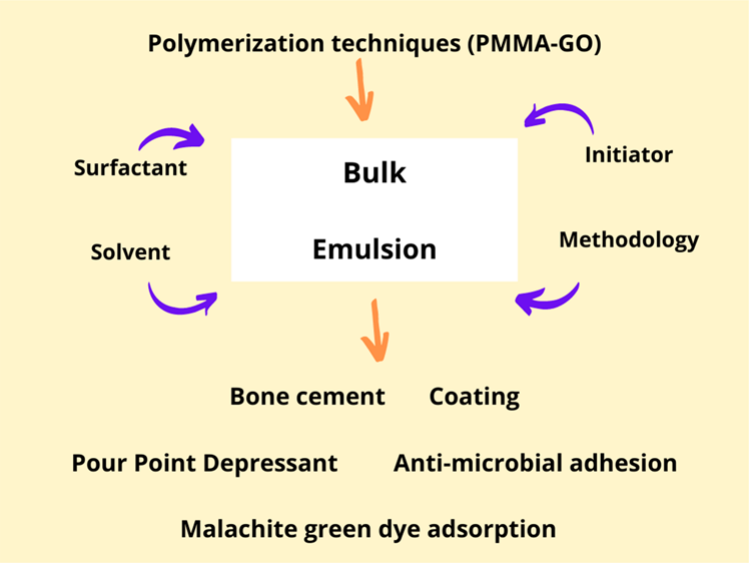

Graphene oxide (GO)-incorporated poly(methyl methacrylate) (PMMA) nanocomposites (PMMA-GO) have demonstrated a wide range of outstanding mechanical, electrical, and physical characteristics. It is of interest to review the synthesis of PMMA-GO nanocomposites and their applications as multifunctional structural materials. The attention of this review is to focus on the radical polymerization techniques, mainly bulk and emulsion polymerization, to prepare PMMA-GO polymeric nanocomposite materials. This review also discusses the effect of solvent polarity on the polymerization process and the types of surfactants (anionic, cationic, nonionic) and initiator used in the polymerization. PMMA-GO nanocomposite synthesis using radical polymerization-based techniques is an active topic of study with several prospects for considerable future improvement and a variety of possible emerging applications. The concentration and dispersity of GO used in the polymerization play critical roles to ensure the functionality and performance of the PMMA-GO nanocomposites.

## Introduction

Polymers of specific performances and specializations often require a hybridization process to produce mutually preferred properties from two or more individual materials. The resulting end properties of the hybrid polymers are summed up from the properties of each component.^1^ A wide selection of organic or inorganic additive materials for producing hybrid polymer composites predominantly relies on the performance needed for a certain application. Hybridization of materials is also often referred to as composite materials, albeit polymer composites can also possess hybrid fillers and additives.^2^ In addition to the proper selection of individual material, the polymerization routes and interactions between the substituents are essential in determining the properties and functionalities of the resultant polymer composites.

Generally, functionalized polymeric hybrid/composite synthesis can be performed via numerous routes and techniques, and these are dependent on the end properties and material applications and the presence of other monomers/materials and solvents. The end properties of the synthesized materials are governed by the polymerization parameter, which in turn contribute to the polymers’ molecular weight, water/solvent solubility, particle morphology and geometry, and surface modification and grafting. During the polymerization process, more than one monomer may be incorporated, and the inclusion of nanoparticles may be performed to acquire polymeric materials with certain properties. This system is often referred to as a nanohybrid material^3^ or, in this context, nanohybrid polymer composites (NHPs). NHPs can be produced by surface modifications of nanoparticles onto the polymer and guarantee enhancement of numerous properties, including increased polymer elastic performance, excellent thermal stability, enhanced wettability, exceptional photostability, electrical conductivity, and eventually improved overall properties and functionalities of the system.^4−7^ Common applications that benefit NHPs are drug delivery systems, polymeric batteries, construction, and architectural and wastewater treatments.

Poly(methyl methacrylate) (PMMA) is one of the widely utilized polymers that is often paired with nanoparticles to produce polymer composites or NHPs. The incorporation of nanoparticles in the PMMA matrix is typically performed to enhance its performance and functionality. PMMA is commonly produced using a free radical initiator and methyl methacrylate (MMA) monomer via a low-cost and hassle-free free radical polymerization. PMMA depolymerizes at 300–400 °C and yields the volatile monomer of MMA.^8^ PMMA can exist in both solid and liquid resin with excellent mechanical properties and optical and thermal stability. These properties, precisely its elastic modulus, thermal stability, flexural strength, fungal resistance, strong UV absorption, and the value of transmittance, can be enhanced when nanoparticles are incorporated into the system.^9−11^

Metal oxide nanoparticles, carbon nanotubes, graphene, and its derivatives can be combined with polymers to produce NHPs.^12^Table 1 tabulates several types of nanoparticles used to produce PMMA nanohybrids for functionalized applications. The nanoparticles listed in Table 1 were incorporated with PMMA to improve their properties by the enhancement of nanocrystallinity, 3D interconnection carbon network, dispersion stability, and band gap value. In this regard, we can acknowledge the incorporation of nanoparticles and PMMA to improve their properties to offer astonishing applications. Specific nanoparticles impart certain targeted properties and performances of the end NHPs such as excellent and tunable optical, electrical, and mechanical properties. In addition to surface functional groups, the surface size and geometry of the nanoparticles enhance their surface activity with the polymer matrix, arising from high surface area of the nanoparticles. Carbon-based nanoparticles are distinguishably known for their outstanding strength, electrical properties, and modular biocompatibility, receiving tremendous interests for functionalized high-end applications. Graphene oxide (GO), like graphene, has a hexagonal carbon structure and yet also possesses hydroxyl groups (−OH), carbonyl groups (C=O), carboxylic acids (single bond COOH), alkoxy groups (C–O–C), and other functional groups with oxygen-based compounds. The presence of −OH makes it soluble in water. The classical Hummers’ method has been commonly used to prepare GO nanoparticles due to its high yield and nontoxicity, safety, and time-efficient procedure.^13^ The oxygenated groups in GO are accountable for numerous benefits over graphene, such as greater solubility and the ability to surface functionalize, which have set off many possibilities for use in NHPs. The functional groups of GO aid its dispersibility in polymeric solutions.^14^ Due to its known hydrophilicity, GO dispersion in hydrophobic polymer matrices needs a significant favorable enthalpic contribution to overcome the polarity disparity, hence the entropy loss necessary for the chain to disperse into the continuum.^15^

**Table 1:** Specializations of PMMA Nanohybrids

polymerization/method	nanoparticle	specialization	ref
free radical of MMA	oxide: SiO_2_, ZnO, and TiO_2_	photoselective nanofilms	(16)
sol–gel of MMA + 3-(trimethoxysilyl)propyl methacrylate (MSMA)	TiO_2_	optoelectronic thin films	(17)
Pickering (oil–water) emulsion	rGO and carbon nanotubes	electrically conductive nanocomposites	(18)
solution casting	CdSe quantum dots	optical characters for LED	(19)

The PMMA nanohybrid demonstrated excellent performance in many specialized applications. Recent findings proved that PMMA-GO nanocomposites were well-suited as a wax inhibitor for Indian waxy crude oil.^20^ The oxidized graphene (GO) possesses oxygenated functional groups on its surface and acts as a possible site for polymerization;^21^ however, the formation of GO agglomerations is commonly expected. The GO agglomeration issue is predominantly caused by surface hydrophilicity, and this phenomenon can be suppressed by the addition of inorganic silica to improve the solubility and dispersibility of graphene sheets and nanoparticles.^22^ A study elaborated their findings on the efficiency of PMMA-GO-based composites on malachite green dye adsorption from water systems. The adsorption was enhanced with the addition of iron(III) oxide, caused by effective collisions between the nanocomposite surfaces and the dye molecules.^23^ This proves that the utilization of types of nanoparticles and additives in PMMA nanohybrids can comprehend targeted polymer functionalization and performances. This review expands various PMMA nanohybrids of GO nanoparticles produced via bulk and emulsion polymerizations for high-performance applications. The selection of main and additive materials and polymerization procedures will be highlighted in this review to correlate these factors with the end properties of the desired polymer nanohybrid composites and its suitability for specific high-performance applications.

## Synthesis Method

### Polymerization of PMMA

**Figure 1:**
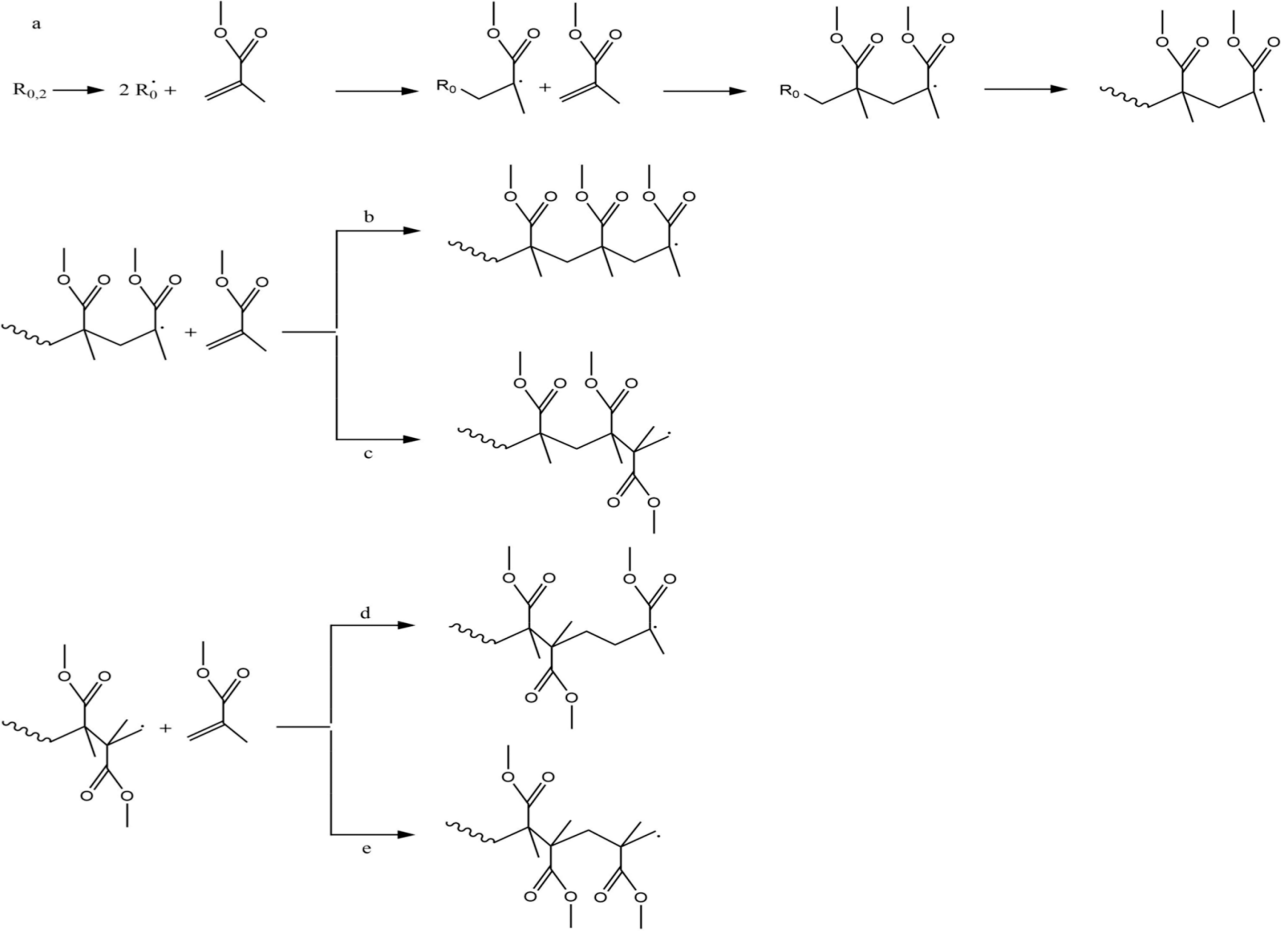
Schematic diagram of free radical polymerization of PMMA: (a) typical reaction of initiator radical formation, chain initiation, and propagation, and (b–e) four types of chain propagation phase. Reprinted with permission from ref (25). Copyright 2020 MDPI (CCBY 4.0).

**Figure 2:**
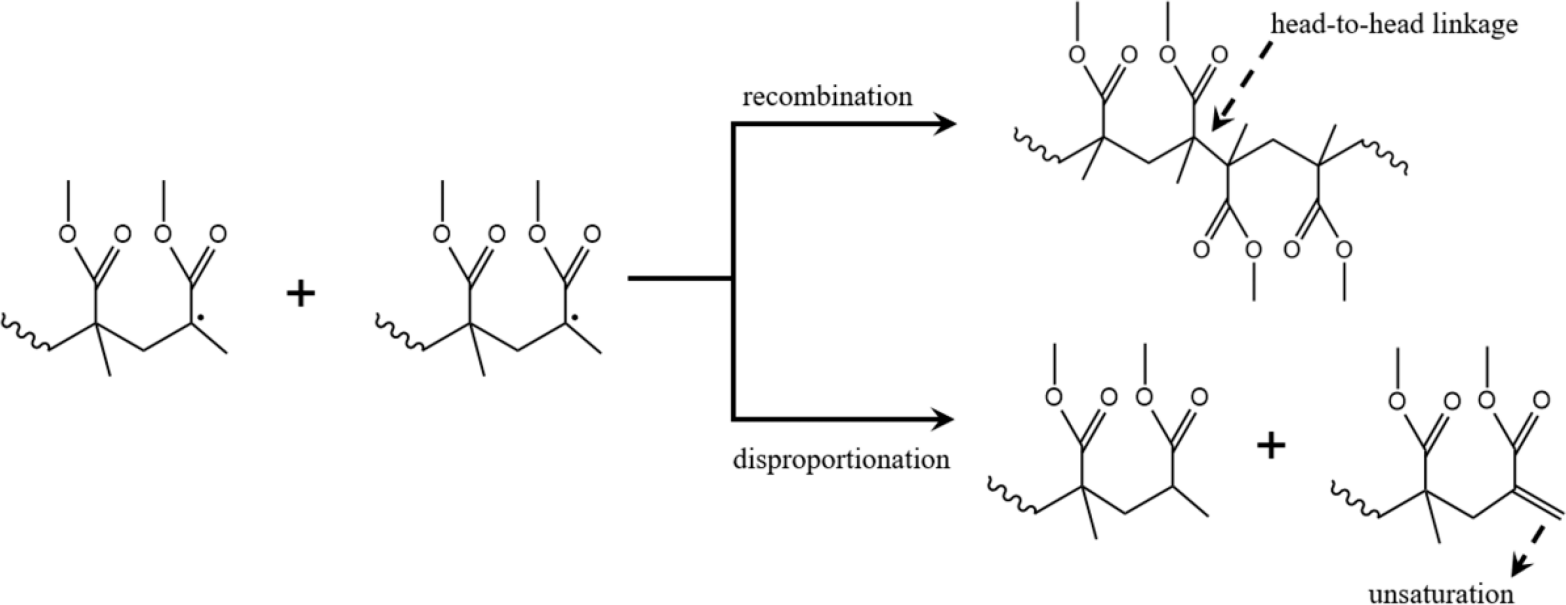
Schematic diagram of chain termination of PMMA radical polymerization via recombination and disproportionation. Reprinted with permission from ref (25). Copyright 2020 MDPI (CCBY 4.0).

**Figure 3:**
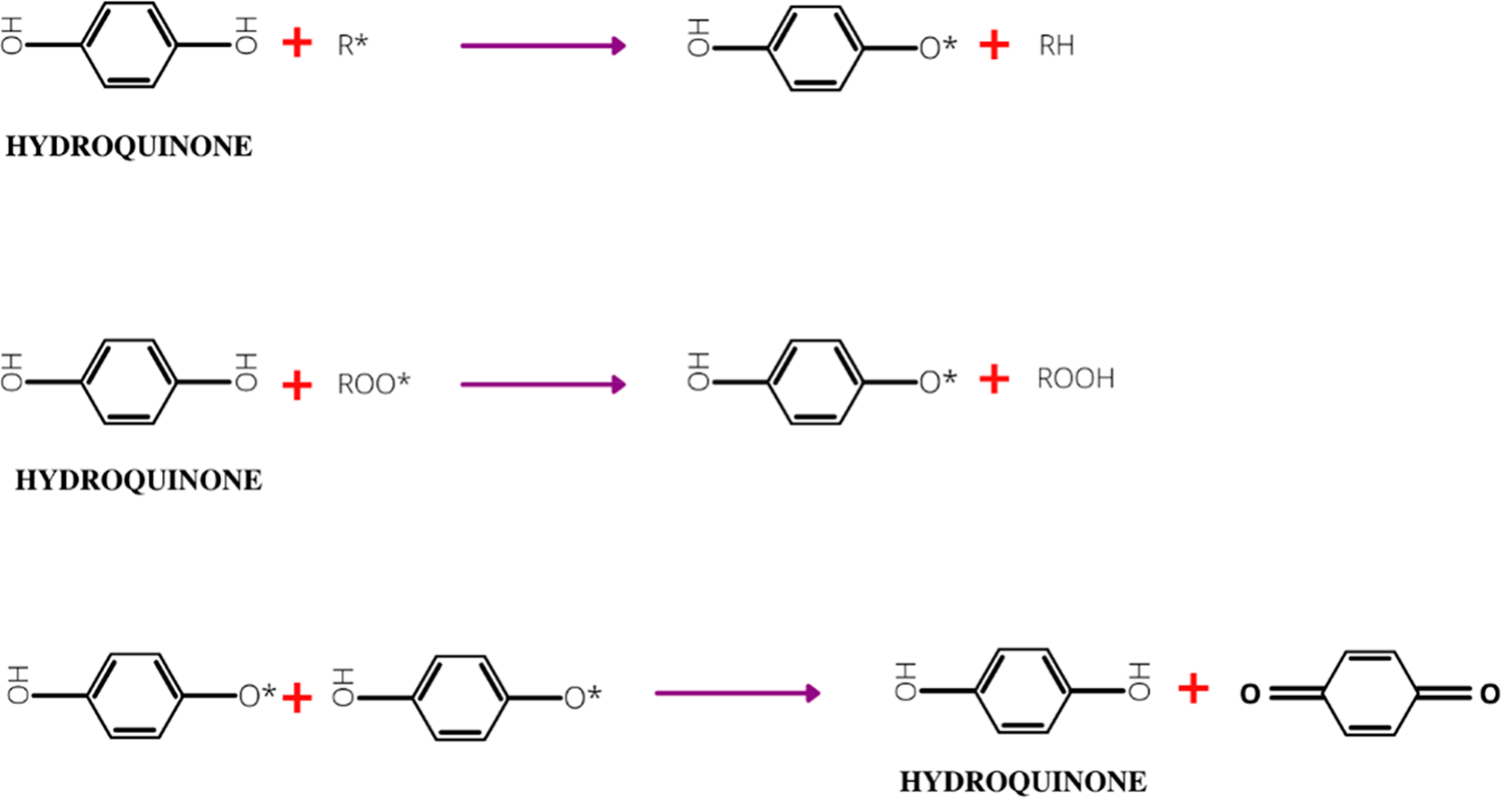
Illustration of the mechanism of hydroquinone as a polymerization inhibitor.

PMMA can be polymerized via a free radical polymerization in a suspension, emulsion, solution, or bulk using MMA as the main monomer and a free radical initiator. The initiator dissociates into free radicals under the influence of energy such as heat or light, depending on the chemical stability of the substances. In Figure 1, the free radicals are denoted as R·, where these radicals react with MMA, forming an oligoradical or an initiation radical chain. At this stage, the process is known as the chain initiation step. The reaction of more MMA monomers with a R-MMA oligoradical produces macroradicals in the propagation step. The active center of the oligoradical is transferred to the new monomer molecules. In this step, the chain continues to grow until all monomers have been used up.^24^ The polymerization ends after the termination step, either by combination or deprotonation. The termination procedure of free radical polymerization disables the active radical centers of the macroradicals by combining the radicals (combination) or transferring the hydrogen atom from the chain to other chains to finally produce one macromolecule.^24^ A study presented the thorough schematic of free radical polymerization of PMMA in Figures 1 and 2.^25^ They also explained four types of propagation phases of MMA: (1) head-to-head; (2) head-to-tail; (3) tail-to-head, and (4) tail-to-tail. The tail substituent is referred to as CH_2_ methylene groups, and the more substituted parts such as CX_1_X_2_ (X_1_ = CH_3_ and X_2_ = COOCH_3_) for MMA correspond to the head part. A free radical scavenger such as hydroquinone (HQ) is extensively used in the free radical polymerization procedure to suppress premature polymerization due to the active free radicals formed due to the temperature built up within the system.^26^ Nevertheless, the excess use of hydroquinone will decrease the polymerization rate.^27^Figure 3 shows the mechanism of HQ as a polymerization inhibitor. The ability of di- and polyhydroxy aromatic compounds is mainly ascribed to their capability to transfer a hydrogen atom to free radicals.^28^ The reaction of the inhibitor and polymerization mixture will form stable compounds in the presence of oxygen.^29^ Hydroquinone has significant advantages in the polymerization of acrylic polymers as opposed to other types of inhibitors due to its versatility including nontoxic, effective use in ambient and elevated polymerization environments, and ease in control during the reaction. Other than hydroquinone, nitroso-based compounds such as 2-methyl-2-nitrosopropane and nitrosobenzene are utilized as inhibitors for acrylic polymers.^30^ Nevertheless, these compounds need a more critical temperature and pressure control to obtain a successful polymerization process. Table 2 shows the use of hydroquinone in various applications for conversion limitation, miscibility, and mechanical strength.

**Table 2:** Hydroquinone in MMA Polymerization

materials	purposes	findings	analytical results	ref
MMA, titanium(III)–salicylaldoxime [Ti(IIII)-SAO], 2.0 wt % HQ	observation on termination of polymerization initiated by the [Ti(IIII)-SAO] redox system	the mechanism of termination was predominantly reciprocal coupling, with a minor contribution from a chain transfer process that involves solvent molecules	polymerization time (90 min):	(27)
			absence of HQ: 80% conversion	
			presence of HQ: 40% conversion	
PMMA, polypyrole dodecylbenzenesulfonate (PPY.DBSA), 1.0 wt % HQ	HQ as compatibilizer agent to increase the dispersibility of PPY.DBSA with PMMA	in the presence of HQ, optical micrographs indicated a moderate amount of phase separation	the thermal events in the blends containing HQ exhibit substantial temperature shifts, indicating more mixing and miscibility than the comparable processes in pure polymers	(32)
PMMA bone cement, rifampin (RIF) 1.00 (1.2 × 10^–3^), HQ 0.13 (1.2 × 10^–3^) g(mol)	characterize the effect of HQ and HQ in RIF incorporation intoPMMA bone cement	the HQ structure of RIF was discovered as the polymerization inhibitor based on the radical scavenging experiment and strength testing	compared to the control, RIF had much lower strength and remained well below 70 MPa for 14 days, and HQ had lowest moduli	(33)
3 wt % of ultrahigh-molecular-weight polyethylene (UHMWPE), BPO, MMA, PMMA, 300 ppm of HQ	improving standard PMMA bone cement’s poor mechanical characteristic	tensile strength value is improved for PMMA bone cement with HQ and BPO compared to the pure PMMA bone cement	tensile strength (MPa):	(34)
			BPO 0.75%, HQ 300 ppm: 49.9 ± 3.75	
			pure PMMA bone cement: 44.5 ± 1.78	

The free radical polymerization procedure also has been extensively performed for surface grafting and functionalization of PMMA with GO. A study revealed that high-density surface grafting of GO can be achieved via controlled radical polymerization of MMA.^21^ The resulting PMMA-GO composites showed an improvement in thermal stability and higher glass transition temperature compared to bare PMMA.^21^ Similar findings on the thermal stability of the GO-functionalized PMMA were reported a few years after, and the average molecular weight and polydispersity index of the molecular weight distribution of the functionalized polymer can be varied by changing the mode of polymerization, either in bulk or in solution.^31^

#### Bulk Polymerization

Bulk polymerization is carried out with a monomer and an initiator being the primary components, without the presence of a solvent. This method entails a simple procedure and is being utilized in the polymerization of step-growth and several types of chain-growth polymers. The bulk polymerization method can be also carried out on various types of monomers at an extensive range of polymerization temperatures.^35^

Based on method 1.1 in Table 3, the bulk polymerization procedure was performed by functionalizing the GO with octadecyl amine (ODA) first and then reacting it with methacryoyl chloride.^36^ Functionalization of GO with ODA significantly improves the dispersion of nanofillers and hence improves the conductivity of polymer nanocomposites.^37^ This reaction is essential to incorporate the polymerizable C=C bonds on the surfaces of the nanoparticles.^36^ As a result, this procedure improves the monomer mixing in organic solvents and obtains covalently bonded PMMA-GO nanocomposites. This is proven by observing the enhancement of thermal stability of PMMA-GO compared to that of the neat PMMA in method 1.1.^36^ Furthermore, certain nanoparticles contain reactive functional groups like −COOH and −OH, which can enhance polymer decomposition or generate covalent bonds, resulting in thermal degradation acceleration.^38^ The thermal degradation of the composites can also be improved by producing a homogeneous dispersion of GO nanosheets, which is achievable in method 1.1. A study of PMMA-functionalized graphene oxide (FGO) also supports the functionality of GO nanosheets to improve the composite thermal stability with a thermal degradation rate (%/°C) difference of 9.8% compared to that of pure PMMA.^39^ In Table 3 method 1.2, it is revealed that GO increased the starting point of temperature for thermal degradation activity. This phenomenon is due to the high molecular weight of the synthesized polymer. However, the study in method 1.1 did not determine the correlation of molecular weight with the improvement of the thermal property of PMMA-GO.

**Table 3:** Polymerization Procedure of Bulk Polymerization of PMMA-GO

1.1	method	1.2
graphite powder, ODA, hexane, acetone, anhydrous toluene, methacryloyl chloride, MMA, AIBN, THF	materials	graphite powder, DMF, BPO, hydroquinone, methanol, MMA
1. preparation of GO (Hummers’ method)	stages	1. preparation of GO (Hummers’ method)
2. reaction of methacryloyl chloride with GO-ODA		2. preparation of the initial monomer/GO mixtures
3. preparation of PMMA-graphene nanocomposites		3. synthesis of PMMA/GO nanocomposites
reaction in a round-bottom flask (magnetic stirrer inside); underwent two freeze–pump–thaw cycles	polymerization setup	ultrasonication for dispersion of GO in solution; nitrogen flow for the reaction of mixture with initiator (polymerization in small test tubes); place into temperature bath for preheated reaction
the products were mixed in THF as a solvent and precipitated in hexane, with drying in vacuum overnight and recovering the samples	procedure to retrieve the sample	stop the polymerization reaction with hydroquinone
		the products were mixed in dichloromethane as a solvent and precipitated in methanol

Referring to Table 3, the apparent points for bulk polymerization of PMMA-GO are ultrasonication for better dispersity of GO in organic compounds, the use of hydroquinone to halt the polymerization, and the functionalization of GO with other materials for polymerization and dispersity. The ultrasonication process is understood to increase the dispersity and homogeneity of GO within the system, while proper sonication time and control can minimize the damage on GO surfaces.^40^ Sonication involves agitating the dispersing particles within a solution using sound waves. A physical vibration is also created, which can break apart substances when the electrical signal is converted. Thus, this causes solutions to mix, resulting in an accelerated dissolution of solids into liquids.

#### Emulsion Polymerization

**Figure 4:**
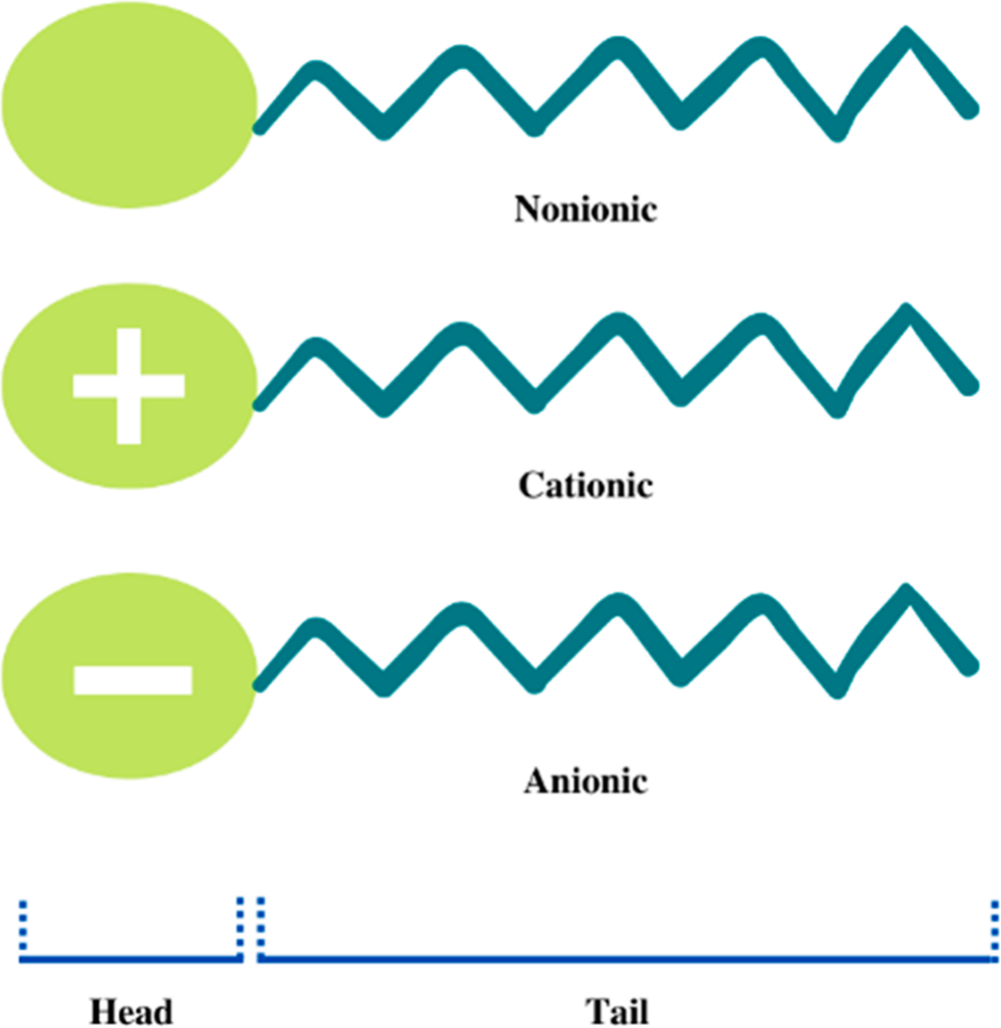
Types of surfactants.

Emulsion polymerization is a process by which free radicals are propagated by monomers dispersed in an aqueous phase. This technique requires the use of an emulsifier to emulsify hydrophobic vinyl polymers via the aqueous phase using an amphiphilic emulsifier of a polar head and nonpolar long-chain tail.^41^ The two main functions of surfactants in emulsion polymerization include controlling the particle size and stabilizing the latex at high solid contents.^42^Table 4 compares several types of emulsifiers or surface-active agents in the polymerization of PMMA. The process also entails the emulsification of hydrophobic monomers with the oil-in-water emulsifier, followed by the reaction initiation using a water-soluble or insoluble initiator. Cationic, anionic, and nonionic surfactants are those with positively, negatively, and uncharged polar head groups, respectively, as shown in Figure 4.

**Table 4:** Surface-Active Agents Used in Emulsion Polymerization of PMMA

surface-active agent	type	ref
polyoxyethylene nonyl phenyl ether	nonionic	(55)
*N*′-hexadecyl-*N*,*N*-dimethylacetamidinium bicarbonate and *N*′-dodecyl-*N*,*N*-dimethylacetamidinium bicarbonate	cationic	(56)
*p*-(11-acrylamido)undecanoyloxyphenyl dimethylsulfonium methyl sulfate (AUPDS)	cationic	(44)
inulin lauryl carbamate (INUTEC SP1)	nonionic	(57)
sodium dodecyl sulfate (SDS)	anionic	(47)
sorbitan trioleate (Span 85)	nonionic	(52)

**Figure 5:**
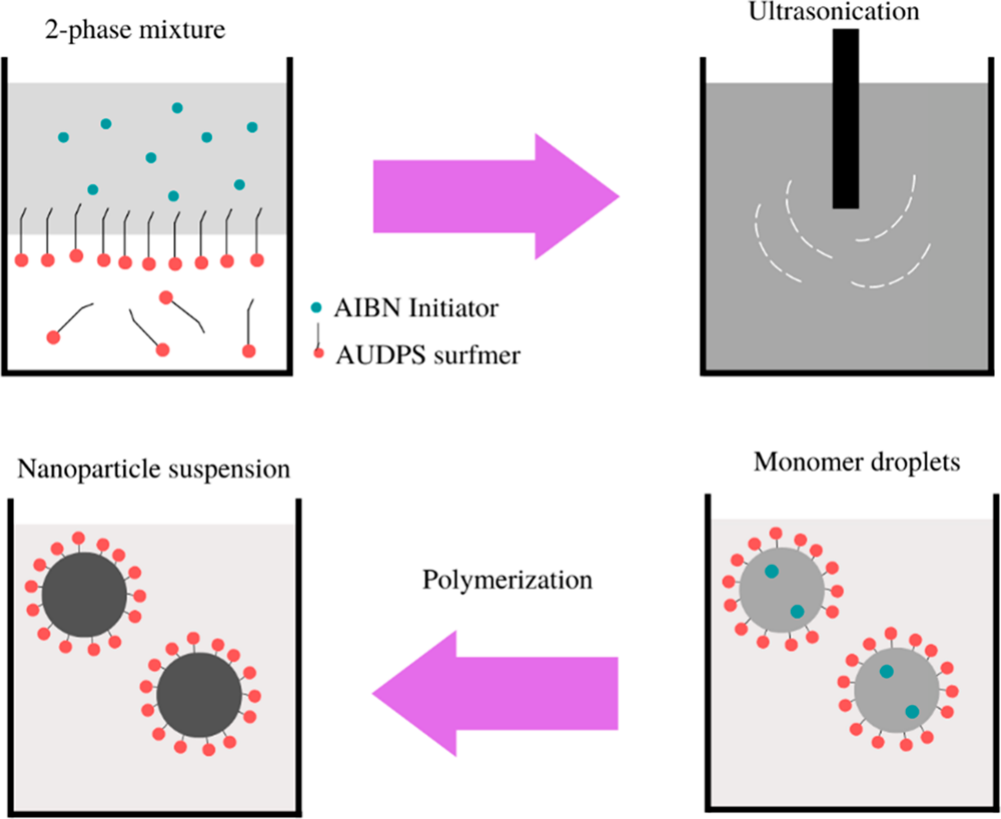
Representation of emulsion polymerization of PMMA using AIBN initiator and AUPDS surfmer (surface active agent). Adapted from ref (43). Copyright 2018 MDPI (CCBY 4.0).

Figure 5 shows emulsion polymerization of PMMA using the AIBN initiator and AUPDS surfmer (surface-active agent).^43^ The use of AUDPS as a cationic surfactant was shown that the cross-linker amounts increased with the AUPDS concentration ratios, and the obtained NPs’ zeta-potential decreased, probably because the AUPDS was internalized during polymerization.^44^ An electrostatic interaction involved sodium dodecyl sulfate (SDS) and a polymer in which anionic head groups are attracted to the oxygen atom polymer that is partially positively charged.^45^ When these surfactants react with water, they form negatively charged anions.^46^ As the concentration of SDS increases, the particle size could rapidly decrease prior to reaching the critical micelle concentration (CMC).^47^ Over time, the particle size decreased relatively slowly with an increase in SDS concentration from the CMC to the critical stability concentration (CSC).^47^ The CSC refers to the lowest surfactant concentration which can produce nanoparticles of the smallest size and greatest stability.^48^ In the presence of a CSC level of SDS concentration, particle size dropped to a minimum level and remained at that size level.^49^ According to eq 1, the number of polymer particles is quantitatively influenced by the rate of radical generation and the concentration of surfactants:^47^
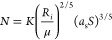
1where *N* is the number of particles, *R*_*i*_ is the rate of radical generation, *a*_s_ in the interfacial surface area, *S* is the surfactant concentration, and μ is the rate of volume increase of polymer particle. As a result, the surfactant and its concentrations have a substantial impact on the particle size.^47^ Nonionic surfactants also have a benefit over other ionic surfactants in that they may produce surfactants with a vast scope of hydrophile–lipophile balance (HLB) by modifying molecular structures, particularly the hydrophilic moiety.^50^ Span 85 is a nonionic surfactant with an oil-soluble characteristic.^51^ The physicochemical properties of Span 85 would be useful in avoiding the following two potential phenomena: concurrent emulsion polymerization of MMA in micelles developed by self-assembled water-soluble surfactants in the dispersed water phase and electrostatic ion complexation with negatively charged PMMA particles.^52^

Based on the previous study, surfactants are capable of affecting the dispersity and solubility of emulsion solution.^53^ In the study, it was shown that vinyl benzyl trimethylammonium chloride (VBTAC) can remain the GO in the monomer phase of MMA. Sodium dodecyl benzenesulfonate (SDBS) occupies both water and monomer phases. SDBS is an anionic surfactant, while VBTAC is a cationic surfactant. VBTAC alters the hydrophilic GO to organophilic GO. As a result, MMA possesses reactive acrylic groups that allow further combination with GO during miniemulsion polymerization.

As shown in Table 5, AIBN and KPS are the examples of initiators used in emulsion polymerization. One of the techniques in emulsion polymerization is Pickering emulsion. In this emulsion reaction, solid particles act as a barrier, preventing the droplets from coalescing when they attach to the interfacial of the emulsion droplets.^54^

**Table 5:** Polymerization Procedure of Emulsion Polymerization of PMMA-GO

2.1	method	2.2
graphite powder, xylene, MMA, AIBN, water	materials	graphite powder, MMA, KPS, deionized water
1. preparation of GO (Hummers’ method)	stages	1. preparation of GO (Hummers’ method)
2. preparation aqueous GO		2. preparation aqueous GO
3. Pickering emulsion polymerization of PMMA-GO		3. Pickering emulsion polymerization of PMMA-GO
high-power sonicator was used to emulsify the mixture; reaction in a glass flask (under stirring)	polymerization setup	sonication of GO and MMA in solution; the flask was vacuum purged before being flushed using nitrogen; polymerization reaction was conducted in the flask
to separate the nanocomposite powder, the resulting slurry was filtered and dried (60 °C)	procedure to retrieve the sample	products were dried in a vacuum at 60 °C

Table 5 shows the emulsion polymerization using methods that are relatively convenient compared to the methods used in the bulk polymerization procedure, where these two methods employ the same procedure using water as the carrying solvent. The main aspect that can be the focus here is the preparation of aqueous GO before mixing it with other materials. Previous studies utilized the preparation of aqueous GO before the polymerization to obtain better dispersion during the polymerization and to avoid any formation of GO agglomerations.^58−61^

### Materials Polarity and Solubility in Polymerization

The selection of polymerization solvent is predominantly dependent on the chemical structure and polarity of the polymers. Solvents are suitable for polymers if their solubility parameters are similar.^62^ Thus, both heterogeneous and homogeneous molecules interact at nearly the same energy level, which promotes the polymer’s solubility.^63^ The solvent must be precisely chosen to prevent chain transfer reactions, which may impede polymer growth.^64^ Thus, the initiator, monomer, and resultant polymer should be soluble in the solvent or solvent blend of interest. Swelling and dissolution will not occur if the chemical structure of both the polymer and solvent molecule differs considerably in polarity.^63^ The chain transfer phenomenon will significantly impact the properties and characteristics of the end polymers such as molecular weight, thermal stability, dielectric constant, and polydispersity index (PDI). According to Floury’s theory, the size of the polymer chains as well as the solvent molecules can affect the excluded volume of polymers and have significant effects on the gel point as well as the gelation process.^65^Table 6 compares several types of solvents used in the free radical polymerization of PMMA-GO. Solvent quality is categorized under good, theta, and poor solvent. A good solvent defines as a solvent that maximizes the monomer–solvent interaction with good chain expansion.^66^ Solvent that creates balanced interaction with the result of an unperturbed chain is categorized under theta solvent.^67^ The poor solvent is the interaction of monomer–solvent is at a minimum level, thus causing chain collapse, precipitation, and clustering.^68^ For predicting the solubility of polymers in organic solvents, Hildebrand’s solubility parameter (δ_H_) has proven to be useful.^69^ Solvents and solutes that have very similar solubility parameters indicate that these compounds are miscible.^69^ The parameter δ_H_ in eq 2 is defined as the square root of the cohesive energy density of compounds, CED = Δ*H*_vap_ – *RT*:
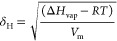
2where Δ*H*_vap_ and *V*_m_ are the enthalpy of vaporization and the molar volume, respectively. Nonpolar substances have a lower δ_H_ value, while polar chemicals have a higher δ_H_ value.^69^

**Table 6:** Types of Solvent and Initiator in Polymerization of PMMA-GO

polymerization technique	solvent	polarity of solvent	initiator	solubility of initiator	ref
Pickering emulsion	water	polar	KPS	polar	(82)
Pickering emulsion	water	polar	KPS	polar	(83)
emulsion	water	polar	KPS	polar	(84)
in situ free radical	toluene	nonpolar	BPO	nonpolar	(79)
in situ free radical	toluene	nonpolar	BPO	nonpolar	(20)
in situ	DMF	polar			(85)
	methanol	polar	AIBN	soluble in the most solvent, but insoluble in water	(86)
dispersion	methanol	polar	AIBN		(87)
bulk radical	acetonitrile and methanol	polar	AIBN		(15)

Generally, the initiator functions to initiate the polymerization of the monomer(s). The polymerization process will begin with the initiator via decomposition to produce primary free radicals.^70,71^ Besides, the molecular weight can be controlled by varying the concentration of the initiator.^70^ The polarity and solubility of the initiator are crucial as other materials undergo polymerization.

#### Polar Solvents in Polymerization of PMMA-GO

Polar solvents are composed of atoms of vast difference in electronegativity, resulting in high probability stabilization of ionic entities through intermolecular interaction.^72^ In a colloidal scale, the length of electrostatic interactions between substances of surface charges can be quantified via the Bjerrum length, λ_B_, and the λ_B_ values for polar solvents are typically small (∼0.7 nm for water).^72^ Short λ_B_ translates to the ability of charged species to interact easily with other polar entities, leading to enhanced interactions. Polar solvents can produce stable and homogeneous colloidal dispersion of graphene oxide due to the electrostatic repulsion between the negatively charged graphene oxide sheets.^73^ The charge is formed from the functional groups containing oxygen that are covalently linked to the surface of graphene. Because oxygen-containing groups are present, polar and hydrogen bonding component values are higher in GO.^74^ Thus, GO can be soluble in water, dimethylformamide (DMF), and methanol. Despite that theoretically PMMA is insoluble with water, it becomes significantly more hydrophilic when exposed to water, as seen by a decrease in contact angle and substantial contact angle hysteresis.^75^ Water can appear near critical water (NCW) at some point. When water is heated at high temperature and subjected to high pressures, it changes its polarity and hydrogen bonding, as a result of different solvent characteristics.^76^ The dielectric constant and density of NCW are like those of acetone under ambient conditions, and its ionization constant is higher than that of the ambient water.^76^ As we know, acetone is a good solvent for PMMA. Hence, the dissolution and swelling of ionic and organic compounds are enabled by these properties. Unlike water, polar solvents like DMF, methanol, and acetonitrile are soluble with PMMA.^77^

#### Nonpolar Solvents in Polymerization of PMMA-GO

In nonpolar solvents such as toluene, *o*-dichlorobenzene (o-DCB), and chlorobenzene, GO is evidenced to be less dispersed but relatively stable.^74^ In addition to the type and chain length of the polymer, the concentration of the composite compositions also affected the solubility of the polymer-functionalized graphene oxide.^78^ Atom transfer radical polymerization (ATRP) can also be used to functionalize GO with poly(*tert*-butyl acrylate), which would be easily dispersed in toluene at a concentration of 1 mg mL^–1^.^22^ Therefore, the solubility of GO in nonpolar is considerable from the previous studies.^20,78,79^ At room temperature to the boiling point of the solvent, PMMA is miscible with toluene even at normal pressure.^80,81^

### Application of PMMA-GO

#### Bone Cement

Advantages of PMMA are being biologically inert and possessing excellent compressive strength, which makes this versatile polymer a viable material for orthopedic applications.^88^ In bone cement applications, PMMA is made in the form of liquid or powder, and it is used to inject as a filling into the injured vertebra.^89^ The mechanical and biological performance of the PMMA can be enhanced with the incorporation of nanoparticles to produce PMMA nanohybrids. GO has been extensively optimized to expand the functionality of PMMA. A work performed by a group of researchers using GO nanoparticles to aid PMMA performance revealed that the usage of only up to 0.25 wt % of GO can significantly affect the mechanical, bending, compression, fracture, fatigue, and thermal properties as well as physical properties of the composites such as density and porosity (Table 7).^90^

**Table 7:** Applications of PMMA-GO

application	optimum GO (wt %)	aids for application	ref
(a) bone cements	0.10, 0.25	hydroquinone	(90)
(b) coating		ATRP (technique)	(95)
(c) pour point depressant	1.00	xylene	(20)
(d) malachite green dye adsorption	0.5		(23)
(e) antimicrobial adhesion	2	orthocryl resin	(96)

Improvements of 13 and 10% in bending strength and Young’s modulus were reported at a loading volume of 0.25 wt % of GO compared to GO-free PMMA. Changes in the atomic bond and the dihedral angles comprising multibody interactions beyond the nearest atom neighbor of two-body interactions have improved the GO bending properties.^91^ Thus, the matrix interface bonding force increases, which leads the polymer composites to have a high strength of bending resistance.^92^ The bending properties are always related to flexural strength as it shows the capability of materials to withstand the applied bending forces on the materials.^93^ As the flexural strength can be correlated to compressive strength, the improvement in bending strength reflects these properties. The compressive strength was improved by 12.6% compared to the control sample. The flexural and compressive strength relationship is shown in eq 3 as studied by Campos:^94^
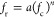
3where *f*_r_ is the flexural strength, *a* is the regression coefficient, *f*_c_ is the compressive strength, and *n* is the power of regression coefficient. The value for *a* is dependent on the similarities of *f*_r_ and *f*_c_ values, where *a* = 1 defines that both flexural and compressive strengths have similar values and are perfectly correlated, and decreasing the *a* value indicates the skewed interdependency between *f*_r_ and *f*_c_ values. The values of *a* and *n* are also dependent on the material properties.^94^

The proper interlocking of the GO and PMMA bone cement matrix is required for the enhancement of its mechanical performance.^90^ Referring to this phenomenon, the nanopowder of GO caused variations in the propagation crack fronts, and this process eventually introduced an off-planeload that formed additional fracture surfaces.^90^ Hence, the study of this application showed an excess of loading GO decreases the fracture toughness, and the optimum value of load GO was 0.1 wt % with the highest value of fracture toughness at 42.2% difference compared to the control sample.

Fatigue is one of the most common causes of catastrophic failure in structural materials, as the materials undergo dynamic crack propagation during cyclic loads. Agglomeration of nanoparticles is prone to occur, which promotes the tendency of fatigue resistance reduction of polymer nanohybrids.^97^ This can be improved by using the proper concentration of GO, ensuring the dispersity and homogeneity of GO within the polymer matrix. Based on a work of crack bridging energy loss mechanisms, the nanomaterials pullout, and fracture at the delamination crack front lower the crack propagation speed.^84^ The fatigue properties result from this application proved that when 0.1% of loading GO was introduced into the PMMA, there was an increase in the mean number of failure cycles (234%) compared to the control sample. While the loading GO of 1.0% exhibited a negative effect on the fatigue performance with the value of −3%.

Bone cement made of PMMA offers many advantages, but thermal bone necrosis can affect other properties. Thus, the thermal stability of bone cement plays a crucial role. The incorporation of nanomaterials into the liquid monomer led to a reduction in the exothermic temperature during polymerization and the thermal necrosis index (TNI), which is very intriguing in ways that inhibit bone thermal necrosis.^98^ The research on this matter showed that from 0.1 to 1.0 wt % GO loading, the residual monomer level increased, but the polymerization heat generated decreased. The carbon-based nanomaterials such as GO have the ability to act as a radical scavenger, which occurs when the cement undergoes polymerization that is attributed to the thermal events. Double bonds within the nanomaterial were converted into reactive species that in turn affected the free radicals while polymerization of cement occurred.^90^

When the loading GO level is high, the dispersion of the nanosized powder, together with the high cement viscosity, can favor air entrapment and promote cement porosity.^90^ It is well acknowledged that an unreacted residual monomer, which is volatile, thus causes porosity within the cement microstructure as a result of its postpolymerization release.^99^ The characterization result shows a reduction value of porosity content (%) when 0.1 wt % loading of GO is introduced with the PMMA compared to the control sample. However, the increase of loading, GO < 0.1 wt %, influences the increase of porosity.

In the synthesis of nanocomposite polymer bone cement, a polymerization inhibitor such as hydroquinone is used to prevent premature polymerization caused by high temperatures or light exposure during storage.^100^ If the polymerization is incomplete, the unreacted monomer may permeate into the surrounding tissues, causing chemical necrosis.^101^

#### Coating

The corrosion resistance of graphene coatings has been demonstrated in numerous studies. Graphene coatings protect substrates by blocking aggressive ions from contacting the surface with the aid of a physical barrier provided by graphene.^102^ This is due to the existence of the passivation layer and the dense filler–matrix interface, making the coating of PMMA with GO capable of preventing corrosion.^103^ Here, we discuss one of the promising previous studies of PMMA-GO for anticorrosive coating applications, as shown in Table 7.

From the study, it is shown that the amount of PMMA in PMMA-grafted GO (PMMA-g-GO) is very crucial as the polymer contributes to the properties of the coating such as hardness, durability, excellent weather resistance, good adhesion to various substrates, and excellent chemical tolerance.^104^ In a system of fixed GO concentration, it was revealed in composites with up to 80% PMMA, the PMMA-g-GO has higher coatings with protection efficiency and η compared to other samples in the absence of PMMA and a lower PMMA concentration. The protection efficiency can be calculated using eq 4:
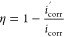
4where the *i*_corr_ is the free corrosion current density for uncoated samples, *i*_corr_^′^ is the free corrosion current density. Even after being immersed for up to 100 h, the η value for a higher fraction of PMMA shows a slight reduction, only from 99.99 to 99.27%, which proves the stability of the coating. Researchers developed photopolymerized PMMA composite coatings containing GO and closely examined the behavior of carboxyl groups that affected the corrosion resistance.^95^ This study demonstrated that the carboxyl group prevented graphene agglomeration, that the acrylic resin dispersion of graphene was improved, and that the barrier properties helped to prevent corrosion.^95^

To produce a uniform coating, the application in Table 7b used the ATRP method in order to synthesize the PMMA-g-GO coating. The ATRP method is one of the most frequently used methods to formulate well-defined polymers with complex morphology. Through ATRP approaches, different triblock copolymer surfaces can be produced that are suited for applications as functional biomaterials and for protective coatings for polar materials.^105^ As a result, a uniform formation of coating can be formed with a desired thickness.^95^ The ATRP reaction is controlled by a balance between propagating radicals and dormant types, predominantly as initiating alkyl halides or macromolecular species.^106^ An ATRP reaction is catalyzed once a transition metal complex reacts with the ATRP initiator to generate radicals and then reacts with the remaining monomer units.^44^ ATRP has been used for coating application because it allows effective control to be obtained over functionalities, molecular weights, well-defined compositions, narrow molecular weight distributions, and architectures.^105^

#### Pour Point Depressant

Crude oil consists of abundant paraffin wax.^107−111^ The formation of wax will disrupt oilfield operations and pipeline transportation.^112,113^ Pour point depressant (PPD) has been used to improve the ability to flow at low temperatures and reduce the wax appearance temperature (WAT).^114^ Polymer nanocomposites are one of the chemical additives that have been used as PPD. The addition of nanoparticles to polymer PMMA could improve its thermal and mechanical properties to maintain favorable flow properties of crude oil treated with PPD.^20^ PMMA-GO is targeted to improve PMMA properties and performs substantially better as a PPD, emphasizing its potential for crude oil transportation applications.^79^ One of the studies in Table 7c shows us the application of PMMA-GO as polymer nanocomposites to be used as PPD.

**Figure 6:**
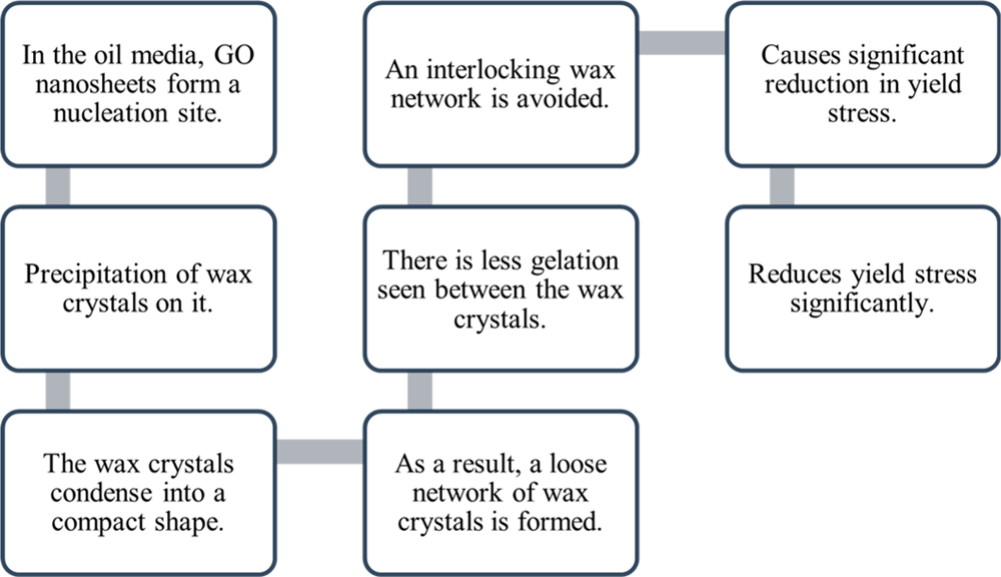
Mechanism by polymer nanocomposite to reduce the wax deposition.

For this application, PMMA with 1 wt % GO was an optimized amount of polymer nanocomposites to be introduced as PPD in the crude oil at concentrations up to 1500 ppm. The WAT was reduced significantly, as low as 15 °C, at a concentration of 1500 ppm compared to that of the nonpolymeric nanocomposite PPD with a reduction as low as 26 °C. As the WAT decreased, the flow ability increased at low temperatures. Crude oil has Newtonian properties above the WAT and non-Newtonian shear thinning characteristics when the temperature drops below the WAT. As a result of the crystallization of wax molecules, the apparent viscosity of a fluid varies inversely with temperature, causing the fluid to flow in a complex manner and show non-Newtonian characteristics.^115^ From the result, it showed that PMMA with 1 wt % GO introduces a huge reduction in viscosity in virgin crude oil at 30 °C from 500 to 3 cP (99.4% viscosity reduction). Even after 15 days with the same additives, it only increases the apparent viscosity by about 33%. Figure 6 explains the mechanism of polymer nanocomposites to reduce wax deposition.^20^ Xylene had been used in this application as a solvent for PMMA-GO to be introduced into virgin crude oil. In order to create improved fluidity of a viscous polymeric nanocomposite, PPD was mixed in xylene prior to doping it in crude oil for pour point measurements.^116^ The xylene-based inhibitor’s wax deposition inhibiting activity is related to its interaction with the developing wax aggregates.^117^ The xylene-based inhibitor has segments that engage with the developing wax crystal and prevent it from growing.^117^

#### Malachite Green Dye Adsorption

Malachite green (MG) dye is a basic dye that is easily soluble in water. The dye is used in the textile industry for wool, paper, cotton, leather, acrylic fibers, silk, and jute, and it also has been used in the aquaculture industry.^23^ Dye adsorption is one of the methods employed to remove the dye in water. Researchers have found that nanocomposites can be used to physically adsorb this dye. Nanomaterials have properties that make them more effective at adsorption than well-known materials, as well as an abundance of active sites making them able to interact with other chemical species.^118^ Due to the disadvantage of nanoparticle agglomeration or sintering that mitigates and reduces their surface area, polymers have gained a great deal of popularity in wastewater control due to their high stability, mechanical strength, high dispersion, and low cost.^119^ Combination of polymeric nanoparticles are more effective at removing pollutants than conventional treatments, and they are nontoxic and less harmful to the environment, as well.^120^ From the research results in Table 7d, they introduce PMMA-GO as nanocomposites for malachite green dye adsorption. A possible mechanism can be explained by the electrostatic interaction between the negative charge of graphene oxide and the positive charge of MG dye, and the exchanged ion between both systems (Table 8).^23^

**Table 8:** Analysis Results for MG Dye Adsorption by PMMA-GO^23^

analysis	results
contact time effect	no significant change after 35 min
pH	increase pH, increase dye removal
initial concentration effect	increase concentration, increase dye removal
temperature	increase temperature, decrease dye removal

When an adsorbent is exposed to a certain concentration of adsorbate, contact time can reflect the adsorption kinetics.^121^ The result in that study shows no significant change after 35 min, which proves that the reaction of PMMA-GO and MG is time-dependent. The contact period of 35 min was sufficient to achieve equilibrium, and the adsorption process was not affected by further contact duration. The increase of pH enhanced the dye removal by PMMA-GO. Activated carbon and deprotonated dye molecules repel each other electrostatically at highly basic conditions, increasing dye adsorption.^122^ It appears that the electrostatic mechanism is applied to this system because activated carbon’s adsorption capacity increased over pH.^123^ The dye removal also increased with increased concentration of MG dye. An absorbent surface’s initial concentration of malachite green dye affects the number of effective collisions between molecules and the absorbent surface.^23^ This is due to the increase of the effective collisions between the PMMA-GO surface and the MG dye molecules.^23^ However, the decrease of dye removal occurs due to the increase of temperature. This behavior indicates that the MG dye adsorption on PMMA-GO was exothermic.^23^

#### Antimicrobial Adhesion

The development of a biofilm begins with the adhesion of microbes to surfaces. All surfaces exposed to an aqueous environment, including implanted biomaterials, soft tissues, tooth surfaces in humans, pipelines in water treatment facilities, rocks in rivers, and ship hulls, can form biofilms.^124^ Various methods have been created over the course of the last few decades to stop microbial adherence to surfaces of biomaterials. Enhancing the surface chemistry of biomaterials is a common component of these methods.^125^ Several factors influence microbial adhesion, including surface energy, hydrophobicity, and hydrophilicity.^126^ One of two approaches is usually used to create antimicrobial-adhesive surfaces for dental biomaterials by applying either hydrophilic or zwitterionic surfaces that generate a hydration layer, and the hydration layer creates a physical and energetic barrier that prevents microbial growth.^127^ It has been reported that nanomaterials derived from carbon, such as graphene oxide nanosheets (nGO), exert antimicrobial properties when in direct contact with microorganisms.^128^ In addition, (functionalized) nGO acts as an antimicrobial-adhesive agent when incorporated into a scaffold, thus improving hydrophilicity.^129^ In order to provide long-lasting antimicrobial effects, pristine nGO was added, which has several hydroxyl (−OH) and carboxyl (−COOH) groups capable of bringing hydrophilicity into PMMA.^96^

Based on research study results presented in Table 7e, the research study focused on incorporating nGO in PMMA to improve antimicrobial adhesion in dentures. As can be observed in Table 9, the optimization of 2 wt % nGO in PMMA shows a very good result compared to other optimizations in the research study. The result of high in hydrophilicity can be understood as increasing roughness and decreasing water contact angles were associated with higher microbial adhesion to PMMA and other biomaterials.^96^ Thus, the 2 wt % nGO shows a very good antimicrobial adhesion effect against *C. albicans*, *E. coli*, *S. aureus*, and *S. mutans*.

**Table 9:** Analysis Results for Antimicrobial Adhesion by nGO Incorporated in PMMA^96^

analysis	results
SEM	surface morphology shows very rough surface compared to other optimizations
water contact angle	highest hydrophilicity
surface hardness	hardest surface
antimicrobial adhesion effect	shows very good effect against *C. albicans*, *E. coli*, *S. aureus*, and *S. mutans*

## Conclusion

The PMMA-GO nanohybrid composite (NHP) is a class of emerging materials with excellent performances and specializations, predominantly caused by the functionalization of GO on the PMMA matrix. The key essential elements in the polymerization of PMMA-GO include surfactant, dispersant, and solvent types to ensure the optimization of targeted PMMA-GO NHP properties and performance. The incorporation of GO in PMMA expands the PMMA potential as a versatile material, generally owing to the main advantages of GO such as having high specific area and excellent mechanical, chemical, and thermal properties. Furthermore, the physicochemical properties, biocompatibility, and bioactive attributes of GO and its composites are modifiable, well-suited for medical-related applications. On the contrary, the utilization of nanofillers such as GO is well-understood to produce a dispersion of a high volume of aggregates due to its surface activity and, as a consequence, can potentially lead to composite failure and deterioration of properties. Nevertheless, the use of appropriate dispersing agents and surfactants may help the end NHP to acquire exceptional anticipated performances. Well-dispersed GO with a suitable concentration in PMMA guarantees the below applications:

i. Bone cement: The inclusion of low loadings (≤0.25 wt %) of GO into a PMMA bone cement can enhance their fatigue properties and particular fracture toughness.
ii. Coatings: The 81% PMMA-*g*-GO coating on copper dramatically increases the corrosion protection and decreases the corrosion.
iii. Pour point depressant: PMMA with 1 wt % GO introduce a huge reduction in viscosity and reduce wax gelation network.
iv. Malachite green dye adsorption: The highest GO incorporated in PMMA has better dye adsorption.
v. Antimicrobial adhesion: The 2 wt % nGO in PMMA guaranteed the antimicrobial adhesion effect.

The importance of the substantial research of GO in the past decades revealed the functionality of GO in vast critical applications, elevating the potential and prospect of traditional materials. This impeccable improvement is not uniquely auspicious on the end materials properties, but also toward sustaining the environment and economy via modular modifications to match specific targets.

## References

[ref1] Zhang W.; Müller A. H. E. Architecture, Self-Assembly and Properties of Well-Defined Hybrid Polymers Based on Polyhedral Oligomeric Silsequioxane (POSS. Prog. Polym. Sci. 2013, 38 (8), 1121–1162. 10.1016/j.progpolymsci.2013.03.002.

[ref2] Swolfs Y.; Gorbatikh L.; Verpoest I. Fibre Hybridisation in Polymer Composites: A Review. Compos. Part A Appl. Sci. Manuf. 2014, 67, 181–200. 10.1016/j.compositesa.2014.08.027.

[ref3] Kaur S.; Gallei M.; Ionescu E. Polymer–Ceramic Nanohybrid Materials. Organice-Inorganic Hybrid Nanomaterials; Sprinter International Publishing: Cham 2014, 267, 143–185. 10.1007/12_2014_282.

[ref4] Corredor L. M.; Husein M. M.; Maini B. B. A Review of Polymer Nanohybrids for Oil Recovery. Adv. Colloid Interface Sci. 2019, 272, 102018 10.1016/j.cis.2019.102018. 31450155

[ref5] Sun J.; Ma Q.; Xue D.; Shan W.; Liu R.; Dong B.; Zhang J.; Wang Z.; Shao B. Polymer/Inorganic Nanohybrids: An Attractive Materials for Analysis and Sensing. TrAC Trends Anal. Chem. 2021, 140, 116273 10.1016/j.trac.2021.116273.

[ref6] Zia J.; Riaz U. Photocatalytic Degradation of Water Pollutants Using Conducting Polymer-Based Nanohybrids: A Review on Recent Trends and Future Prospects. J. Mol. Liq. 2021, 340, 117162 10.1016/j.molliq.2021.117162.

[ref7] Prakash S.; Malhotra M.; Shao W.; Tomaro-Duchesneau C.; Abbasi S. Polymeric Nanohybrids and Functionalized Carbon Nanotubes as Drug Delivery Carriers for Cancer Therapy. Adv. Drug Delivery Rev. 2011, 63 (14–15), 1340–1351. 10.1016/j.addr.2011.06.013. 21756952

[ref8] Manoukian O. S.; Sardashti N.; Stedman T.; Gailiunas K.; Ojha A.; Penalosa A.; Mancuso C.; Hobert M.; Kumbar S. G. Biomaterials for Tissue Engineering and Regenerative Medicine. Encyclopedia of Biomedical Engineering; Elsevier, 2019; pp 462–482, 10.1016/B978-0-12-801238-3.64098-9.

[ref9] Alsaad A. M.; Al-Bataineh Q. M.; Ahmad A. A.; Jum’h I.; Alaqtash N.; Bani-Salameh A. A. Optical Properties of Transparent PMMA-PS/ZnO NPs Polymeric Nanocomposite Films: UV-Shielding Applications. Mater. Res. Express 2019, 6 (12), 126446 10.1088/2053-1591/ab68a0.

[ref10] Giti R.; Firouzmandi M.; Zare Khafri N.; Ansarifard E. Influence of Different Concentrations of Titanium Dioxide and Copper Oxide Nanoparticles on Water Sorption and Solubility of Heat-cured PMMA Denture Base Resin. Clin. Exp. Dent. Res. 2022, 8 (1), 287–293. 10.1002/cre2.527. 35015382 PMC8874036

[ref11] Mangal U.; Kim J.-Y.; Seo J.-Y.; Kwon J.-S.; Choi S.-H. Novel Poly(Methyl Methacrylate) Containing Nanodiamond to Improve the Mechanical Properties and Fungal Resistance. Materials (Basel). 2019, 12 (20), 3438 10.3390/ma12203438. 31640147 PMC6829541

[ref12] Ferreira Soares D. C.; Domingues S. C.; Viana D. B.; Tebaldi M. L. Polymer-Hybrid Nanoparticles: Current Advances in Biomedical Applications. Biomed. Pharmacother. 2020, 131, 110695 10.1016/j.biopha.2020.110695. 32920512

[ref13] Liu X.; Shi L.; Jiang W.; Zhang J.; Huang L. Taking Full Advantage of KMnO4 in Simplified Hummers Method: A Green and One Pot Process for the Fabrication of Alpha MnO2 Nanorods on Graphene Oxide. Chem. Eng. Sci. 2018, 192, 414–421. 10.1016/j.ces.2018.07.044.

[ref14] Woo Y. C.; Kim S.-H.; Shon H. K.; Tijing L. D. Introduction: Membrane Desalination Today, Past, and Future. Current Trends and Future Developments on (Bio-) Membranes; Elsevier, 2019; pp xxv–xlvi, 10.1016/B978-0-12-813551-8.00028-0.

[ref15] JANG J.; KIM M.; JEONG H.; SHIN C. Graphite Oxide/Poly(Methyl Methacrylate) Nanocomposites Prepared by a Novel Method Utilizing Macroazoinitiator. Compos. Sci. Technol. 2009, 69 (2), 186–191. 10.1016/j.compscitech.2008.09.039.

[ref16] El-Bashir S. M.; Al-Harbi F. F.; Elburaih H.; Al-Faifi F.; Yahia I. S. Red Photoluminescent PMMA Nanohybrid Films for Modifying the Spectral Distribution of Solar Radiation inside Greenhouses. Renew. Energy 2016, 85, 928–938. 10.1016/j.renene.2015.07.031.

[ref17] Yuwono A. H.; Liu B.; Xue J.; Wang J.; Elim H. I.; Ji W.; Li Y.; White T. J. Controlling the Crystallinity and Nonlinear Optical Properties of Transparent TiO _2_ – PMMA Nanohybrids. J. Mater. Chem. 2004, 14 (20), 2978–2987. 10.1039/B403530E.

[ref18] Wang B.; Liang P.; Li W.; Gao Y. Electrical Conductivity of Poly(Methyl Methacrylate) Nanocomposites Containing Interconnected Carbon Nanohybrid Network Based on Pickering Emulsion Strategy. Soft Mater. 2021, 19 (4), 468–479. 10.1080/1539445X.2020.1868511.

[ref19] Heiba Z. K.; Mohamed M. B.; Imam N. G. Optical and Structural Characteristics of CdSe/PMMA Nanocomposites. Int. Polym. Process. 2018, 33 (2), 226–233. 10.3139/217.3469.

[ref20] Sharma R.; Mahto V.; Vuthaluru H. Synthesis of PMMA/Modified Graphene Oxide Nanocomposite Pour Point Depressant and Its Effect on the Flow Properties of Indian Waxy Crude Oil. Fuel 2019, 235, 1245–1259. 10.1016/j.fuel.2018.08.125.

[ref21] Kumar M.; Chung J. S.; Hur S. H. Controlled Atom Transfer Radical Polymerization of MMA onto the Surface of High-Density Functionalized Graphene Oxide. Nanoscale Res. Lett. 2014, 9 (1), 345 10.1186/1556-276X-9-345. 25114639 PMC4115242

[ref22] Johnson D. W.; Dobson B. P.; Coleman K. S. A Manufacturing Perspective on Graphene Dispersions. Curr. Opin. Colloid Interface Sci. 2015, 20 (5–6), 367–382. 10.1016/j.cocis.2015.11.004.

[ref23] Rajabi M.; Mahanpoor K.; Moradi O. Preparation of PMMA/GO and PMMA/GO-Fe3O4 Nanocomposites for Malachite Green Dye Adsorption: Kinetic and Thermodynamic Studies. Compos. Part B Eng. 2019, 167, 544–555. 10.1016/j.compositesb.2019.03.030.

[ref24] Lamaoui A.; García-Guzmán J. J.; Amine A.; Palacios-Santander J. M.; Cubillana-Aguilera L. Synthesis Techniques of Molecularly Imprinted Polymer Composites. Molecularly Imprinted Polymer Composites; Elsevier, 2021; pp 49–91, 10.1016/B978-0-12-819952-7.00002-0.

[ref25] Moens E.; De Smit K.; Marien Y.; Trigilio A.; Van Steenberge P.; Van Geem K.; Dubois J.-L.; D’hooge D. Progress in Reaction Mechanisms and Reactor Technologies for Thermochemical Recycling of Poly(Methyl Methacrylate). Polymers (Basel). 2020, 12 (8), 1667 10.3390/polym12081667. 32727004 PMC7464549

[ref26] Banjong P.; Sankham N.; Duangnga W.; Intatha U.; Soykeabkaew N.; Duangphet S. The Modification of Acrylic Denture Base Resin as Materials for Artificial Teeth: Effect of Hydroquinone and Methyl Methacrylate Monomer. ScienceAsia 2020, 46S (1), 97 10.2306/scienceasia1513-1874.2020.S014.

[ref27] Yashoda M. P.; Sherigara B. S.; Nayak P. V.; Venkateswaran G. AQUEOUS POLYMERIZATION OF METHYL METHACRYLATE INITIATED BY TITANIUM(III)—SALICYLALDOXIME REDOX SYSTEM: A KINETIC STUDY. J. Macromol. Sci. Part A 2000, 37 (11), 1487–1505. 10.1081/MA-100101167.

[ref28] Kiokias S.; Varzakas T.; Oreopoulou V. In Vitro Activity of Vitamins, Flavonoids, and Natural Phenolic Antioxidants Against the Oxidative Deterioration of Oil-Based Systems. Crit. Rev. Food Sci. Nutr. 2008, 48 (1), 78–93. 10.1080/10408390601079975. 18274966

[ref29] Yeow J.; Chapman R.; Gormley A. J.; Boyer C. Up in the Air: Oxygen Tolerance in Controlled/Living Radical Polymerisation. Chem. Soc. Rev. 2018, 47 (12), 4357–4387. 10.1039/C7CS00587C. 29718038 PMC9857479

[ref30] Nicolaÿ R.; Mosnáček J.; Kar K. K.; Fruchey S. O.; Cloeter M. D.; Harner R. S.; Matyjaszewski K. Efficient Polymerization Inhibition Systems for Acrylic Acid Distillation: Vapor-Phase Inhibitors. Ind. Eng. Chem. Res. 2012, 51 (12), 4467–4471. 10.1021/ie201709y.

[ref31] Tsagkalias I.; Manios T.; Achilias D. Effect of Graphene Oxide on the Reaction Kinetics of Methyl Methacrylate In Situ Radical Polymerization via the Bulk or Solution Technique. Polymers (Basel). 2017, 9 (9), 432 10.3390/polym9090432. 30965738 PMC6418969

[ref32] Shakoor A.; Foot P. J. S.; Rizvi T. Z. Conductive Poly(Methyl Methacrylate)-Polypyrrole Dodecylbenzenesulfonate (PMMA-PPy.DBSA) Blends Prepared in Solution in the Presence of Hydroquinone. J. Mater. Sci. Mater. Electron. 2010, 21 (12), 1270–1276. 10.1007/s10854-010-0060-8.

[ref33] Funk G. A.; Menuey E. M.; Cole K. A.; Schuman T. P.; Kilway K. V.; McIff T. E. Radical Scavenging of Poly(Methyl Methacrylate) Bone Cement by Rifampin and Clinically Relevant Properties of the Rifampin-Loaded Cement. Bone Joint Res. 2019, 8 (2), 81–89. 10.1302/2046-3758.82.BJR-2018-0170.R2. 30915214 PMC6397418

[ref34] Yang D. H.; Yoon G. H.; Kim S. H.; Rhee J. M.; Kim Y. S.; Khang G. Surface and Chemical Properties of Surface-Modified UHMWPE Powder and Mechanical and Thermal Properties of It Impregnated PMMA Bone Cement, III: Effect of Various Ratios of Initiator/Inhibitor on the Surface Modification of UHMWPE Powder. J. Biomater. Sci. Polym. Ed. 2005, 16 (9), 1121–1138. 10.1163/1568562054798572. 16231603

[ref35] Jašo V.; Stoiljković D.; Radičević R.; Bera O. Kinetic Modeling of Bulk Free-Radical Polymerization of Methyl Methacrylate. Polym. J. 2013, 45 (6), 631–636. 10.1038/pj.2013.6.

[ref36] Pramoda K. P.; Hussain H.; Koh H. M.; Tan H. R.; He C. B. Covalent Bonded Polymer-Graphene Nanocomposites. J. Polym. Sci. Part A Polym. Chem. 2010, 48 (19), 4262–4267. 10.1002/pola.24212.

[ref37] Yuan X. Y.; Zou L. L.; Liao C. C.; Dai J. W. Improved Properties of Chemically Modified Graphene/Poly(Methyl Methacrylate) Nanocomposites via a Facile in-Situ Bulk Polymerization. Express Polym. Lett. 2012, 6 (10), 847–858. 10.3144/expresspolymlett.2012.90.

[ref38] Bikiaris D. Can Nanoparticles Really Enhance Thermal Stability of Polymers? Part II: An Overview on Thermal Decomposition of Polycondensation Polymers. Thermochim. Acta 2011, 523 (1–2), 25–45. 10.1016/j.tca.2011.06.012.

[ref39] Li Y.-L.; Kuan C.-F.; Chen C.-H.; Kuan H.-C.; Yip M.-C.; Chiu S.-L.; Chiang C.-L. Preparation, Thermal Stability and Electrical Properties of PMMA/Functionalized Graphene Oxide Nanosheets Composites. Mater. Chem. Phys. 2012, 134 (2–3), 677–685. 10.1016/j.matchemphys.2012.03.050.

[ref40] Gao Y.; Jing H. W.; Chen S. J.; Du M. R.; Chen W. Q.; Duan W. H. Influence of Ultrasonication on the Dispersion and Enhancing Effect of Graphene Oxide–Carbon Nanotube Hybrid Nanoreinforcement in Cementitious Composite. Compos. Part B Eng. 2019, 164, 45–53. 10.1016/j.compositesb.2018.11.066.

[ref41] El-hoshoudy A. N. M. B. Emulsion Polymerization Mechanism. Recent Research in Polymerization; InTech, 2018; 10.5772/intechopen.72143.

[ref42] Guyot A.; Tauer K. Reactive Surfactants in Emulsion Polymerization. Polymer Synthesis; Springer-Verlag: Berlin, 2005; pp 43–65, 10.1007/BFb0024126.

[ref43] Albernaz V. L.; Bach M.; Weber A.; Southan A.; Tovar G. E. M. Active Ester Containing Surfmer for One-Stage Polymer Nanoparticle Surface Functionalization in Mini-Emulsion Polymerization. Polymers (Basel). 2018, 10 (4), 408 10.3390/polym10040408. 30966443 PMC6415249

[ref44] Sunitha K.; Reghunadhan Nair C. P. Synthetic Applications of Click Chemistry in Thermosetting Block and Graft Polymers. Handbook of Thermoset Plastics; Elsevier, 2022; pp 931–952, 10.1016/B978-0-12-821632-3.00002-6.

[ref45] Taylor D. J. F.; Thomas R. K.; Penfold J. Polymer/Surfactant Interactions at the Air/Water Interface. Adv. Colloid Interface Sci. 2007, 132 (2), 69–110. 10.1016/j.cis.2007.01.002. 17328859

[ref46] Livingstone R. A.; Nagata Y.; Bonn M.; Backus E. H. G. Two Types of Water at the Water–Surfactant Interface Revealed by Time-Resolved Vibrational Spectroscopy. J. Am. Chem. Soc. 2015, 137 (47), 14912–14919. 10.1021/jacs.5b07845. 26544087

[ref47] Guo Z.; Liu J.; Li Y.; Lin H.; Wang H.; Tam K. C.; Liu G. Effects of Dispersion Techniques on the Emulsion Polymerization of Methyl Methacrylate. Colloid Polym. Sci. 2021, 299 (7), 1147–1159. 10.1007/s00396-021-04835-4.

[ref48] Magrì D.; Sánchez-Moreno P.; Caputo G.; Gatto F.; Veronesi M.; Bardi G.; Catelani T.; Guarnieri D.; Athanassiou A.; Pompa P. P.; Fragouli D. Laser Ablation as a Versatile Tool To Mimic Polyethylene Terephthalate Nanoplastic Pollutants: Characterization and Toxicology Assessment. ACS Nano 2018, 12 (8), 7690–7700. 10.1021/acsnano.8b01331. 29944342

[ref49] Wang H.; Pan Q.; Rempel G. L. Micellar Nucleation Differential Microemulsion Polymerization. Eur. Polym. J. 2011, 47 (5), 973–980. 10.1016/j.eurpolymj.2010.11.009.

[ref50] Aramaki K.; Kunieda H.; Ishitobi M. Self-Organization of Sucrose Fatty Acid Ester in Water. Proceedings of the International Conference on Colloid and Surface Science, 25th Anniversary of the Division of Colloid and Surface Chemistry, The Chemical Society of Japan; Studies in Surface Science and Catalysis; Elsevier 2001, 132, 985–988. 10.1016/S0167-2991(01)82250-3.

[ref51] Abdulkarim M. F.; Abdullah G. Z.; Sakeena M. H. F.; Chitneni M.; Yam M. F.; Mahdi E. S.; Salman I. M.; Ameer O. Z.; Sattar M. A.; Basri M.; Noor A. M. Study of Pseudoternary Phase Diagram Behaviour and the Effect of Several Tweens and Spans on Palm Oil Esters Characteristics. Int. J. Drug Delivery 2011, 3 (1), 95–100. 10.5138/ijdd.2010.0975.0215.03058.

[ref52] Watanabe T.; Karita K.; Ono T. Flow Synthesis of Monodisperse Micron-Sized Polymer Particles by Heterogeneous Polymerization Using a Water-in-Oil Slug Flow with a Non-Ionic Surfactant. Colloid Polym. Sci. 2020, 298 (9), 1273–1281. 10.1007/s00396-020-04705-5.

[ref53] Wang H.; Wang L.; Meng S.; Lin H.; Correll M.; Tong Z. Nanocomposite of Graphene Oxide Encapsulated in Polymethylmethacrylate (PMMA): Pre-Modification, Synthesis, and Latex Stability. J. Compos. Sci. 2020, 4 (3), 118 10.3390/jcs4030118.

[ref54] Bon S. A. F. Chapter 4. Pickering Suspension, Mini-Emulsion and Emulsion Polymerization. Particle-Stabilized Emulsions and Colloids: Formation and Applications; RSC, 2014; pp 65–92, 10.1039/9781782620143-00065.

[ref55] Matsusaka N.; Suzuki T.; Okubo M. Effect of Partitioning of Monomer and Emulsifier in Aqueous Media on Particle Formation in Emulsion Homopolymerization of Hydrophobic and Hydrophilic Monomers with a Nonionic Emulsifier. Polym. J. 2013, 45 (2), 153–159. 10.1038/pj.2012.109.

[ref56] Fowler C. I.; Muchemu C. M.; Miller R. E.; Phan L.; O’Neill C.; Jessop P. G.; Cunningham M. F. Emulsion Polymerization of Styrene and Methyl Methacrylate Using Cationic Switchable Surfactants. Macromolecules 2011, 44 (8), 2501–2509. 10.1021/ma102936a.

[ref57] Nestor J.; Esquena J.; Solans C.; Levecke B.; Booten K.; Tadros T. F. Emulsion Polymerization of Styrene and Methyl Methacrylate Using a Hydrophobically Modified Inulin and Comparison with Other Surfactants. Langmuir 2005, 21 (11), 4837–4841. 10.1021/la047018y. 15896021

[ref58] Lee S.; Lim S.; Lim E.; Lee K. K. Synthesis of Aqueous Dispersion of Graphenes via Reduction of Graphite Oxide in the Solution of Conductive Polymer. J. Phys. Chem. Solids 2010, 71 (4), 483–486. 10.1016/j.jpcs.2009.12.017.

[ref59] Zu S.-Z.; Han B.-H. Aqueous Dispersion of Graphene Sheets Stabilized by Pluronic Copolymers: Formation of Supramolecular Hydrogel. J. Phys. Chem. C 2009, 113 (31), 13651–13657. 10.1021/jp9035887.

[ref60] Arzac A.; Leal G. P.; Fajgar R.; Tomovska R. Comparison of the Emulsion Mixing and In Situ Polymerization Techniques for Synthesis of Water-Borne Reduced Graphene Oxide/Polymer Composites: Advantages and Drawbacks. Part. Part. Syst. Charact. 2014, 31 (1), 143–151. 10.1002/ppsc.201300286.

[ref61] Lan Y.; Caciagli A.; Guidetti G.; Yu Z.; Liu J.; Johansen V. E.; Kamp M.; Abell C.; Vignolini S.; Scherman O. A.; Eiser E. Unexpected Stability of Aqueous Dispersions of Raspberry-like Colloids. Nat. Commun. 2018, 9 (1), 3614 10.1038/s41467-018-05560-3. 30190497 PMC6127241

[ref62] Dimian A. C.; Bildea C. S.; Kiss A. A. Chemical Product Design. Computer Aided Chemical Engineering; Elsevier 2014, 35, 489–523. 10.1016/B978-0-444-62700-1.00012-7.

[ref63] Senichev V. Y.; Tereshatov V. V. Simple Solvent Characteristics. Handbook of Solvents; Elsevier, 2014; pp 117–149, 10.1016/B978-1-895198-64-5.50006-4.

[ref64] Argenta D. F.; dos Santos T. C.; Campos A. M.; Caon T. Hydrogel Nanocomposite Systems. Nanocarriers for Drug Delivery; Elsevier, 2019; pp 81–131, 10.1016/B978-0-12-814033-8.00003-5.

[ref65] Li Y.; Huang Q.; Shi T.; An L. How Does Solvent Molecular Size Affect the Microscopic Structure in Polymer Solutions?. J. Chem. Phys. 2006, 125 (4), 044902 10.1063/1.2213610. 16942187

[ref66] Holehouse A. S.; Pappu R. V. Collapse Transitions of Proteins and the Interplay Among Backbone, Sidechain, and Solvent Interactions. Annu. Rev. Biophys. 2018, 47 (1), 19–39. 10.1146/annurev-biophys-070317-032838. 29345991 PMC10740066

[ref67] Katsumoto Y. Amphiphilic, Thermoresponsive Polymers Interacting with Explicit Solvent. Molecular Basics of Liquids and Liquid-Based Materials; Springer: Singapore, 2021; pp 337–361, 10.1007/978-981-16-5395-7_11.

[ref68] Ensafi A. A.; Kazemifard N.; Saberi Dehkordi Z. Parameters That Affect Molecular Imprinting Polymers. Molecularly Imprinted Polymer Composites; Elsevier, 2021; pp 21–48, 10.1016/B978-0-12-819952-7.00010-X.

[ref69] Ueno K.; Fukai T.; Nagatsuka T.; Yasuda T.; Watanabe M. Solubility of Poly(Methyl Methacrylate) in Ionic Liquids in Relation to Solvent Parameters. Langmuir 2014, 30 (11), 3228–3235. 10.1021/la404797g. 24568265

[ref70] Su W.-F. Radical Chain Polymerization. Principles of Polymer Design and Synthesis; Springer, 2013; pp 137–183, 10.1007/978-3-642-38730-2_7.

[ref71] Wan Ibrahim W. H. B.; Mujtaba I. M. Dynamic Optimization of Solution Polymerization Process of Methyl Methacrylate in Batch Reactors. Computer-Aided Chemical Engineering; Elsevier, 2012; pp 1326–1330, 10.1016/B978-0-444-59506-5.50096-1.

[ref72] Zeegers-Huyskens T.; Huyskens P. Intermolecular Forces. Intermolecular Forces; Springer: Berlin, 1991; pp 1–30, 10.1007/978-3-642-76260-4_1.

[ref73] Akbari A.; Meragawi S. E.; Martin S. T.; Corry B.; Shamsaei E.; Easton C. D.; Bhattacharyya D.; Majumder M. Solvent Transport Behavior of Shear Aligned Graphene Oxide Membranes and Implications in Organic Solvent Nanofiltration. ACS Appl. Mater. Interfaces 2018, 10 (2), 2067–2074. 10.1021/acsami.7b11777. 29251906 PMC10707417

[ref74] Konios D.; Stylianakis M. M.; Stratakis E.; Kymakis E. Dispersion Behaviour of Graphene Oxide and Reduced Graphene Oxide. J. Colloid Interface Sci. 2014, 430, 108–112. 10.1016/j.jcis.2014.05.033. 24998061

[ref75] Bettencourt A.; Almeida A. J. Poly(Methyl Methacrylate) Particulate Carriers in Drug Delivery. J. Microencapsul. 2012, 29 (4), 353–367. 10.3109/02652048.2011.651500. 22251239

[ref76] Erdmenger T.; Guerrero-Sanchez C.; Vitz J.; Hoogenboom R.; Schubert U. S. Recent Developments in the Utilization of Green Solvents in Polymer Chemistry. Chem. Soc. Rev. 2010, 39 (8), 3317 10.1039/b909964f. 20601997

[ref77] Klapper M.; Nenov S.; Haschick R.; Müller K.; Müllen K. Oil-in-Oil Emulsions: A Unique Tool for the Formation of Polymer Nanoparticles. Acc. Chem. Res. 2008, 41 (9), 1190–1201. 10.1021/ar8001206. 18759463

[ref78] Lee S. H.; Dreyer D. R.; An J.; Velamakanni A.; Piner R. D.; Park S.; Zhu Y.; Kim S. O.; Bielawski C. W.; Ruoff R. S. Polymer Brushes via Controlled, Surface-Initiated Atom Transfer Radical Polymerization (ATRP) from Graphene Oxide. Macromol. Rapid Commun. 2010, 31 (3), 281–288. 10.1002/marc.200900641. 21590903

[ref79] Al-Sabagh A. M.; Betiha M. A.; Osman D. I.; Hashim A. I.; El-Sukkary M. M.; Mahmoud T. Preparation and Evaluation of Poly(Methyl Methacrylate)-Graphene Oxide Nanohybrid Polymers as Pour Point Depressants and Flow Improvers for Waxy Crude Oil. Energy Fuels 2016, 30 (9), 7610–7621. 10.1021/acs.energyfuels.6b01105.

[ref80] Jouault N.; Kumar S. K.; Smalley R. J.; Chi C.; Moneta R.; Wood B.; Salerno H.; Melnichenko Y. B.; He L.; Guise W. E.; Hammouda B.; Crawford M. K. Do Very Small POSS Nanoparticles Perturb S-PMMA Chain Conformations?. Macromolecules 2018, 51 (14), 5278–5293. 10.1021/acs.macromol.8b00432.

[ref81] Belsey K. E.; Topping C.; Farrand L. D.; Holder S. J. Inhibiting the Thermal Gelation of Copolymer Stabilized Nonaqueous Dispersions and the Synthesis of Full Color PMMA Particles. Langmuir 2016, 32 (11), 2556–2566. 10.1021/acs.langmuir.6b00063. 26927952

[ref82] Min T. H.; Choi H. J. Synthesis of Poly(Methyl Methacrylate)/Graphene Oxide Nanocomposite Particles via Pickering Emulsion Polymerization and Their Viscous Response under an Electric Field. Macromol. Res. 2017, 25 (6), 565–571. 10.1007/s13233-017-5109-6.

[ref83] Huang Y.; Wang X.; Jin X.; Wang T. Study on the PMMA/GO Nanocomposites with Good Thermal Stability Prepared by in Situ Pickering Emulsion Polymerization. J. Therm. Anal. Calorim. 2014, 117 (2), 755–763. 10.1007/s10973-014-3794-3.

[ref84] Wen-Ping W.; Cai-Yuan P. Preparation and Characterization of Poly(Methyl Methacrylate)-Intercalated Graphite Oxide/Poly(Methyl Methacrylate) Nanocomposite. Polym. Eng. Sci. 2004, 44 (12), 2335–2339. 10.1002/pen.20261.

[ref85] Feng L.; Guan G.; Li C.; Zhang D.; Xiao Y.; Zheng L.; Zhu W. *In Situ* Synthesis of Poly(Methyl Methacrylate)/Graphene Oxide Nanocomposites Using Thermal-Initiated and Graphene Oxide-Initiated Polymerization. J. Macromol. Sci. Part A 2013, 50 (7), 720–727. 10.1080/10601325.2013.792217.

[ref86] Rajabi M.; Mahanpoor K.; Moradi O. Thermodynamic and Kinetic Studies of Crystal Violet Dye Adsorption with Poly(Methyl Methacrylate)–Graphene Oxide and Poly(Methyl Methacrylate)–Graphene Oxide–Zinc Oxide Nanocomposites. J. Appl. Polym. Sci. 2019, 47495 10.1002/app.47495.

[ref87] Zhang K.; Zhang W. L.; Choi H. J. Facile Fabrication of Self-Assembled PMMA/Graphene Oxide Composite Particles and Their Electroresponsive Properties. Colloid Polym. Sci. 2013, 291 (4), 955–962. 10.1007/s00396-012-2814-8.

[ref88] Chen W.-J.; Kao Y.-H.; Yang S.-C.; Yu S.-W.; Tu Y.-K.; Chung K.-C. Impact of Cement Leakage Into Disks on the Development of Adjacent Vertebral Compression Fractures. J. Spinal Disord. Technol. 2010, 23 (1), 35–39. 10.1097/BSD.0b013e3181981843. 20065868

[ref89] Baroud G.; Vant C.; Giannitsios D.; Bohner M.; Steffen T. Effect of Vertebral Shell on Injection Pressure and Intravertebral Pressure in Vertebroplasty. Spine (Phila. Pa. 1976). 2005, 30 (1), 68–74. 10.1097/01.brs.0000149188.51154.5b. 15626984

[ref90] Paz E.; Forriol F.; del Real J. C.; Dunne N. Graphene Oxide versus Graphene for Optimisation of PMMA Bone Cement for Orthopaedic Applications. Mater. Sci. Eng., C 2017, 77, 1003–1011. 10.1016/j.msec.2017.03.269. 28531971

[ref91] Poulin P.; Jalili R.; Neri W.; Nallet F.; Divoux T.; Colin A.; Aboutalebi S. H.; Wallace G.; Zakri C. Superflexibility of Graphene Oxide. Proc. Natl. Acad. Sci. U. S. A. 2016, 113 (40), 11088–11093. 10.1073/pnas.1605121113. 27647890 PMC5056031

[ref92] Zhang H.; Gao X.; Li H.; Wu W.; Sun S. Effect of Graphene Oxide on Tensile and Flexural Properties of Carbon/Glass Hybrid Fiber-reinforced Polymer Composite. Polym. Compos. 2021, 42 (10), 5348–5360. 10.1002/pc.26228.

[ref93] Azammi A. M. N.; Ilyas R. A.; Sapuan S. M.; Ibrahim R.; Atikah M. S. N.; Asrofi M.; Atiqah A. Characterization Studies of Biopolymeric Matrix and Cellulose Fibres Based Composites Related to Functionalized Fibre-Matrix Interface. Interfaces in Particle and Fibre Reinforced Composites; Elsevier, 2020; pp 29–93, 10.1016/B978-0-08-102665-6.00003-0.

[ref94] Campos R.; Larrain M. M. M.; Zaman M.; Pozadas V. Relationships between Compressive and Flexural Strengths of Concrete Based on Fresh Field Properties. Int. J. Pavement Res. Technol. 2021, 14 (2), 161–167. 10.1007/s42947-020-1074-0.

[ref95] Qi K.; Sun Y.; Duan H.; Guo X. A Corrosion-Protective Coating Based on a Solution-Processable Polymer-Grafted Graphene Oxide Nanocomposite. Corros. Sci. 2015, 98, 500–506. 10.1016/j.corsci.2015.05.056.

[ref96] Lee J.-H.; Jo J.-K.; Kim D.-A.; Patel K. D.; Kim H.-W.; Lee H.-H. Nano-Graphene Oxide Incorporated into PMMA Resin to Prevent Microbial Adhesion. Dent. Mater. 2018, 34 (4), e63–e72. 10.1016/j.dental.2018.01.019. 29402540

[ref97] Borrego L. P.; Costa J. D. M.; Ferreira J. A. M.; Silva H. Fatigue Behaviour of Glass Fibre Reinforced Epoxy Composites Enhanced with Nanoparticles. Compos. Part B Eng. 2014, 62, 65–72. 10.1016/j.compositesb.2014.02.016.

[ref98] Ormsby R.; McNally T.; Mitchell C.; Halley P.; Martin D.; Nicholson T.; Dunne N. Effect of MWCNT Addition on the Thermal and Rheological Properties of Polymethyl Methacrylate Bone Cement. Carbon N. Y. 2011, 49 (9), 2893–2904. 10.1016/j.carbon.2011.02.063.

[ref99] Gonçalves G.; Cruz S. M. A.; Ramalho A.; Grácio J.; Marques P. A. A. P. Graphene Oxide versus Functionalized Carbon Nanotubes as a Reinforcing Agent in a PMMA/HA Bone Cement. Nanoscale 2012, 4 (9), 2937 10.1039/c2nr30303e. 22499394

[ref100] Ranjan R. K.; Kumar M.; Kumar R.; Ali M. F. Bone Cement. Int. J. Orthop. Sci. 2017, 3 (4b), 79–82. 10.22271/ortho.2017.v3.i4b.12.

[ref101] DEB S.; KOLLER G. Acrylic Bone Cement: Genesis and Evolution. Orthopaedic Bone Cements; Elsevier, 2008; pp 167–182, 10.1533/9781845695170.2.167.

[ref102] Ollik K.; Lieder M. Review of the Application of Graphene-Based Coatings as Anticorrosion Layers. Coatings 2020, 10 (9), 883 10.3390/coatings10090883.

[ref103] Aravindh S.; Karthikeyan B. Graphene Oxide—Polymethyl Methacrylate Coatings for Corrosion Protection of Aerospace Aluminium Alloy 7075—T651 Surfaces. Eng. Res. Express 2020, 2 (3), 035034 10.1088/2631-8695/abb4f3.

[ref104] Maiti T. K.; Parvate S.; Pragya; Singh J.; Dixit P.; Bhuvanesh E.; Vennapusa J. R.; Chattopadhyay S. Plastics in Coating Applications. Reference Module in Materials Science and Materials Engineering; Elsevier, 2021; 10.1016/B978-0-12-820352-1.00176-0.

[ref105] Król P.; Chmielarz P. Recent Advances in ATRP Methods in Relation to the Synthesis of Copolymer Coating Materials. Prog. Org. Coatings 2014, 77 (5), 913–948. 10.1016/j.porgcoat.2014.01.027.

[ref106] Matyjaszewski K. Atom Transfer Radical Polymerization (ATRP): Current Status and Future Perspectives. Macromolecules 2012, 45 (10), 4015–4039. 10.1021/ma3001719.

[ref107] França D.; Coutinho D. M.; Barra T. A.; Xavier R. S.; Azevedo D. A. Molecular-Level Characterization of Brazilian Pre-Salt Crude Oils by Advanced Analytical Techniques. Fuel 2021, 293, 120474 10.1016/j.fuel.2021.120474.

[ref108] França D.; Pereira V. B.; Coutinho D. M.; Ainstein L. M.; Azevedo D. A. Speciation and Quantification of High Molecular Weight Paraffins in Brazilian Whole Crude Oils Using High-Temperature Comprehensive Two-Dimensional Gas Chromatography. Fuel 2018, 234, 1154–1164. 10.1016/j.fuel.2018.07.145.

[ref109] Zhao J.; Zhao W.; Dong H.; Wei L.; Liu Y. New Approach for the In Situ Microscopic Observation of Wax Crystals in Waxy Crude Oil during Quiescent and Dynamic Cooling. ACS Omega 2020, 5 (20), 11491–11506. 10.1021/acsomega.0c00606. 32478238 PMC7254814

[ref110] Eke W. I.; Achugasim O.; Ofordile S. E.; Ajienka J. A.; Akaranta O. Influence of Heavy Organics Composition on Wax Properties and Flow Behavior of Waxy Crude Oils. Chem. Sci. Int. J. 2019, 1–12. 10.9734/CSJI/2019/v27i230109.

[ref111] Rehan M.; Nizami A.-S.; Taylan O.; Al-Sasi B. O.; Demirbas A. Determination of Wax Content in Crude Oil. Pet. Sci. Technol. 2016, 34 (9), 799–804. 10.1080/10916466.2016.1169287.

[ref112] Aiyejina A.; Chakrabarti D. P.; Pilgrim A.; Sastry M. K. S. Wax Formation in Oil Pipelines: A Critical Review. Int. J. Multiph. Flow 2011, 37 (7), 671–694. 10.1016/j.ijmultiphaseflow.2011.02.007.

[ref113] Chala G. T.; Sulaiman S. A.; Japper-Jaafar A. Flow Start-up and Transportation of Waxy Crude Oil in Pipelines-A Review. J. Nonnewton. Fluid Mech. 2018, 251, 69–87. 10.1016/j.jnnfm.2017.11.008.

[ref114] Elganidi I.; Elarbe B.; Abdullah N.; Ridzuan N. Synthesis of a Novel Terpolymer of (BA-Co-SMA-Co-MA) as Pour Point Depressants to Improve the Flowability of the Malaysian Crude Oil. Mater. Today Proc. 2021, 42, 28–32. 10.1016/j.matpr.2020.08.628.

[ref115] Deka B.; Sharma R.; Mandal A.; Mahto V. Synthesis and Evaluation of Oleic Acid Based Polymeric Additive as Pour Point Depressant to Improve Flow Properties of Indian Waxy Crude Oil. J. Pet. Sci. Eng. 2018, 170, 105–111. 10.1016/j.petrol.2018.06.053.

[ref116] Sharma R.; Deka B.; Mahto V.; Vuthaluru H.; Li C.-Z. Investigation into the Flow Assurance of Waxy Crude Oil by Application of Graphene-Based Novel Nanocomposite Pour Point Depressants. Energy Fuels 2019, 33 (12), 12330–12345. 10.1021/acs.energyfuels.9b03124.

[ref117] Akinyemi O. P.; Udonne J. D.; Efeovbokhan V. E.; Ayoola A. A. A Study on the Use of Plant Seed Oils, Triethanolamine and Xylene as Flow Improvers of Nigerian Waxy Crude Oil. J. Appl. Res. Technol. 2016, 14 (3), 195–205. 10.1016/j.jart.2016.04.006.

[ref118] Osagie C.; Othmani A.; Ghosh S.; Malloum A.; Kashitarash Esfahani Z.; Ahmadi S. Dyes Adsorption from Aqueous Media through the Nanotechnology: A Review. J. Mater. Res. Technol. 2021, 14, 2195–2218. 10.1016/j.jmrt.2021.07.085.

[ref119] Pant H. R.; Kim H. J.; Joshi M. K.; Pant B.; Park C. H.; Kim J. I.; Hui K. S.; Kim C. S. One-Step Fabrication of Multifunctional Composite Polyurethane Spider-Web-like Nanofibrous Membrane for Water Purification. J. Hazard. Mater. 2014, 264, 25–33. 10.1016/j.jhazmat.2013.10.066. 24269971

[ref120] Lazaridis N. K.; Bakoyannakis D. N.; Deliyanni E. A. Chromium(VI) Sorptive Removal from Aqueous Solutions by Nanocrystalline Akaganèite. Chemosphere 2005, 58 (1), 65–73. 10.1016/j.chemosphere.2004.09.007. 15522334

[ref121] Cano F. Kinetics and Thermodynamics of Lead Adsorption from Aqueous Solutions Onto Iranian Sepiolite and Zeolite. Int. J. Environ. Res. 2015, 9 (3), 1001–1010. 10.22059/IJER.2015.988.

[ref122] Jung K.-W.; Choi B. H.; Hwang M.-J.; Jeong T.-U.; Ahn K.-H. Fabrication of Granular Activated Carbons Derived from Spent Coffee Grounds by Entrapment in Calcium Alginate Beads for Adsorption of Acid Orange 7 and Methylene Blue. Bioresour. Technol. 2016, 219, 185–195. 10.1016/j.biortech.2016.07.098. 27494099

[ref123] Liu Q.-S.; Zheng T.; Wang P.; Jiang J.-P.; Li N. Adsorption Isotherm, Kinetic and Mechanism Studies of Some Substituted Phenols on Activated Carbon Fibers. Chem. Eng. J. 2010, 157 (2–3), 348–356. 10.1016/j.cej.2009.11.013.

[ref124] Busscher H. J.; van der Mei H. C. Microbial Adhesion in Flow Displacement Systems. Clin. Microbiol. Rev. 2006, 19 (1), 127–141. 10.1128/CMR.19.1.127-141.2006. 16418527 PMC1360269

[ref125] Delaviz Y.; Santerre J. P.; Cvitkovitch D. G. Infection Resistant Biomaterials. Biomaterials and Medical Device - Associated Infections; Elsevier, 2015; pp 223–254, 10.1533/9780857097224.2.223.

[ref126] Cai L.; Wu D.; Xia J.; Shi H.; Kim H. Influence of Physicochemical Surface Properties on the Adhesion of Bacteria onto Four Types of Plastics. Sci. Total Environ. 2019, 671, 1101–1107. 10.1016/j.scitotenv.2019.03.434.

[ref127] Kim K.-I.; Kim D.-A.; Patel K. D.; Shin U. S.; Kim H.-W.; Lee J.-H.; Lee H.-H. Carbon Nanotube Incorporation in PMMA to Prevent Microbial Adhesion. Sci. Rep. 2019, 9 (1), 4921 10.1038/s41598-019-41381-0. 30894673 PMC6427005

[ref128] Mejias Carpio I. E.; Mangadlao J. D.; Nguyen H. N.; Advincula R. C.; Rodrigues D. F. Graphene Oxide Functionalized with Ethylenediamine Triacetic Acid for Heavy Metal Adsorption and Anti-Microbial Applications. Carbon N. Y. 2014, 77, 289–301. 10.1016/j.carbon.2014.05.032.

[ref129] Jain V. P.; Chaudhary S.; Sharma D.; Dabas N.; Lalji R. S. K.; Singh B. K.; Jaiswar G. Advanced Functionalized Nanographene Oxide as a Biomedical Agent for Drug Delivery and Anti-Cancerous Therapy: A Review. Eur. Polym. J. 2021, 142, 110124 10.1016/j.eurpolymj.2020.110124.

